# Data on true tRNA diversity among uncultured and bacterial strains

**DOI:** 10.1016/j.dib.2016.04.049

**Published:** 2016-04-26

**Authors:** Bhagwan N. Rekadwad, Chandrahasya N. Khobragade

**Affiliations:** School of Life Sciences, Swami Ramanand Teerth Marathwada University, Nanded, Maharashtra 431606, India

**Keywords:** GC content, RNA signatures, tRNA types, true RNA, Uncultured archaea and bacteria

## Abstract

Complete genome sequences of two uncultured archaea (BX649197 and CR937008) and 10 uncultured bacteria (AC160099, FP245538-FP245540, FP312972, FP312974-75, FP312977, FP312985 and NZ_JPJG01000067) were used for creation of digital data of tRNA. tRNAscan-SE and ENDMEMO GC calculating tools were used for detection of tRNA, drawing their structures and calculation of GC percent. Seven archaeal and 48 bacterial tRNA were detected from above 12 sequences. Four archaeal and 30 bacterial tRNA showed cove score more than 20% are called as true tRNA. Three tRNA of uncultured bacteria (AC160099) has the presence of the variable loop. The tRNA of FP245540, FP245575, FP245577 and FP245585 has one variable loop each. The true tRNA of archaea were Alanine, Arginine and Cysteine-type tRNA, while the majority of bacteria true tRNA classified as Alanine, Glutamic acid, Isoleucine, Leucine, Methionine, Phenylalanine, Proline and Valine-type tRNA with cove score ranged from 70% to 97.15%. Archaeal and bacterial have GC content approximately 43% and 34.7–63.3% respectively. Archaeal tRNA has 60.4–64.2% GC content. Similarly, bacterial tRNA contributed 49.3–66.3% GC content to the total GC content. This generated data is useful for studies on diversity of tRNA among prokaryotes.

**Specifications Table**

**Table:** 

Subject area	*Microbiology*
More specific subject area	*Bioinformatics, Extremophiles, Environmental Microbiology and Biotechnology, Microbial diversity informatics*
Type of data	*Table, images*
How data was acquired	*Through genomic databases, tRNAscan-SE and ENDMEMO GC calculating tools*
Data format	*Analyzed and filtered*
Experimental factors	*Complete genome sequences were used for generation of data on tRNA*
Experimental features	*Complete genome used for analysis of tRNA*
Data source location	*School of Life Sciences, S. R. T. M. University, Nanded (India)*
Data accessibility	Data is within this article.

**Value of the data**

• Analysis for the presence of tRNA using complete DNA sequences has generated novel data on tRNA diversity among the uncultured archaea and bacteria.
• Data on GC content of newly detected tRNA appears to be white snow for research on tRNA.
• Data on digitization and GC content of tRNA were carried out first time by us and made available to users.

## 1 Data

Complete DNA sequences of two uncultured archaea (BX649197 and CR937008) and 10 uncultured bacteria (AC160099, FP245538-FP245540, FP312972, FP312974-75, FP312977, FP312985 and NZ_JPJG01000067) was obtained through NCBI׳s BioSample database. Presence of tRNA and its data was acquired through tRNAscan-SE tool. Detected tRNA were classified in different type on the basis of amino acid coded and cove score. ENDMEMO GC calculating tool generated data on GC content in percentage (Supplementary files 1, 2 and 3). See also *NCBI repository* (http://www.ncbi.nlm.nih.gov/nuccore).

## 2 Experimental design, materials and methods

Complete DNA sequences in FASTA format were retrieved from NCBI BioSample database. The data in the form of tRNA structure was generated using tRNAScan-SE tool. The tRNA was classified into different types based on the amino acid code and cove score [1], [2], [3]. The data on GC percent [4] both complete genome and for identified/detected tRNA in the complete was acquired through ENDMEMO GC calculating tool. ENDMEMO GC plotting tool used draw pattern of GC distribution graphical representations. Upper and lower red line indicate maximum and minimum GC percentage, while middle blue line indicate average GC percentage distributed in DNA sequence [5].
