# Supplementary material for: Data on true tRNA diversity among uncultured and bacterial strains
Source: Data Brief. 2016 Apr 26;7:1538–40. doi: 10.1016/j.dib.2016.04.049 (PMC4865659; doi:10.1016/j.dib.2016.04.049)
Supplement: Supplementary file 1 — Supplementary material [file mmc1.docx]

**Table 1**

Results of Analysis of tRNA detected in uncultured archaea genomes using tRNAScan-SE tool

| **Uncultured archaeon clone fos0128g3+03e1 (CR937008)** |
| --- |
| Sequence tRNA Bounds tRNA Anti Intron Bounds Cove  Name tRNA # Begin End Type Codon Begin End Score  -------- ------ ---- ------ ---- ----- ----- ---- ------   \| gi\|82617204\|emb\|CR937008.1\| 1 55744 55673 Ala TGC 0 0 75.25 \| \| --- \| \| Top of Form  Bottom of Form \| \| gi\|82617204\|emb\|CR937008.1\| 2 52463 52391 Cys GCA 0 0 54.34 \| \| Top of Form  Bottom of Form \| \| gi\|82617204\|emb\|CR937008.1\| 3 24734 24663 Arg CCG 0 0 60.10 \| \| Top of Form  Bottom of Form \|   **Run Statistics:**  Started: Thu Mar 10 03:44:16 PST 2016  ------------------------------------------------------------  Search Mode: Archaeal  Searching with: tRNAscan + EufindtRNA -> Cove  Covariance model: TRNA2-arch.cm  tRNAscan parameters: Strict  EufindtRNA parameters: Relaxed (Int Cutoff= -36)  ------------------------------------------------------------  First-pass (tRNAscan/EufindtRNA) Stats:  ---------------  Sequences read: 1  Seqs w/at least 1 hit: 1  Bases read: 66432 (x2 for both strands)  Bases in tRNAs: 854  tRNAs predicted: 6  Av. tRNA length: 142  Script CPU time: 0.01 s  Scan CPU time: 0.04 s  Scan speed: 3321.6 Kbp/sec  First pass search(es) ended: Thu Mar 10 03:44:16 PST 2016  Cove Stats:  -----------  Candidate tRNAs read: 6  Cove-confirmed tRNAs: 3  Bases scanned by covels: 938  % seq scanned by covels: 0.7 %  Script CPU time: 0.01 s  Cove CPU time: 1.78 s  Scan speed: 527.0 bp/sec  Cove analysis of tRNAs ended: Thu Mar 10 03:44:18 PST 2016  Summary  --------  Overall scan speed: 72208.7 bp/sec  tRNAs decoding Standard 20 AA: 3  Selenocysteine tRNAs (TCA): 0  Possible suppressor tRNAs (CTA,TTA): 0  tRNAs with undetermined/unknown isotypes: 0  Predicted pseudogenes: 0  -------  Total tRNAs: 3  tRNAs with introns: 0  \|  Isotype / Anticodon Counts:  Ala : 1 AGC: GGC: CGC: TGC: 1  Gly : 0 ACC: GCC: CCC: TCC:  Pro : 0 AGG: GGG: CGG: TGG:  Thr : 0 AGT: GGT: CGT: TGT:  Val : 0 AAC: GAC: CAC: TAC:  Ser : 0 AGA: GGA: CGA: TGA: ACT: GCT:  Arg : 1 ACG: GCG: CCG: 1 TCG: CCT: TCT:  Leu : 0 AAG: GAG: CAG: TAG: CAA: TAA:  Phe : 0 AAA: GAA:  Asn : 0 ATT: GTT:  Lys : 0 CTT: TTT:  Asp : 0 ATC: GTC:  Glu : 0 CTC: TTC:  His : 0 ATG: GTG:  Gln : 0 CTG: TTG:  Ile : 0 AAT: GAT: TAT:  Met : 0 CAT:  Tyr : 0 ATA: GTA:  Supres: 0 CTA: TTA:  Cys : 1 ACA: GCA: 1  Trp : 0 CCA:  SelCys: 0 TCA:  **Predicted tRNA Secondary Structures:**  gi\|82617204\|emb\|CR937008.1\|.trna1 (55744-55673) Length: 72 bp  Type: Ala Anticodon: TGC at 33-35 (55712-55710) Score: 75.25  * \| * \| * \| * \| * \| * \| * \|  Seq: GGGCTCGTAGATCAGTGGAAGATCGTCGCCTTTGCGAGGCGAAGGcCCTGGGTTCAAATCCCAGCGAGTCCA  Str: >>>>>>>..>>>>.......<<<<.>>>>>.......<<<<<.....>>>>>.......<<<<<<<<<<<<.  gi\|82617204\|emb\|CR937008.1\|.trna2 (52463-52391) Length: 73 bp  Type: Cys Anticodon: GCA at 34-36 (52430-52428) Score: 54.34  * \| * \| * \| * \| * \| * \| * \|  Seq: GCCAAGGTGGCGGAGAGGCaCACGCGGCTGACTGCAGATCAGCTATaCCCCGGTTCAAATCCGGGCCTTGGCT  Str: >>>>>>>..>>>..........<<<.>>>>>.......<<<<<.....>>>>>.......<<<<<<<<<<<<.  gi\|82617204\|emb\|CR937008.1\|.trna3 (24734-24663) Length: 72 bp  Type: Arg Anticodon: CCG at 34-36 (24701-24699) Score: 60.10  * \| * \| * \| * \| * \| * \| * \|  Seq: GGGGTCGTAGGGTAGGGGAtATCCTGTCAGGTTCCGGACCTGACGACCTGGGTTCGAATCCCAGCGACTCCG  Str: >>>>>>>..>>>..........<<<.>>>>>.......<<<<<....>>>>>.......<<<<<<<<<<<<.  **Candidate tRNA Predictions in BED format:**  gi\|82617204\|emb\|CR937008.1\| 55672 55744 gi\|82617204\|emb\|CR937008.1\|.tRNA1-AlaTGC 752 -  gi\|82617204\|emb\|CR937008.1\| 52390 52463 gi\|82617204\|emb\|CR937008.1\|.tRNA2-CysGCA 543 -  gi\|82617204\|emb\|CR937008.1\| 24662 24734 gi\|82617204\|emb\|CR937008.1\|.tRNA3-ArgCCG 601 - |
| **Uncultured archaeon clone 0418F12 (BX649197)** |
| Sequence tRNA Bounds tRNA Anti Intron Bounds Cove  Name tRNA # Begin End Type Codon Begin End Score  -------- ------ ---- ------ ---- ----- ----- ---- ------   \| gi\|40217425\|emb\|BX649197.1\| 1 18270 18341 Ala CGC 0 0 75.97 \| \| --- \| \| Top of Form  Bottom of Form \|   **Run Statistics:**  Started: Thu Mar 10 03:46:18 PST 2016  ------------------------------------------------------------  Search Mode: Archaeal  Searching with: tRNAscan + EufindtRNA -> Cove  Covariance model: TRNA2-arch.cm  tRNAscan parameters: Strict  EufindtRNA parameters: Relaxed (Int Cutoff= -36)  ------------------------------------------------------------  First-pass (tRNAscan/EufindtRNA) Stats:  ---------------  Sequences read: 1  Seqs w/at least 1 hit: 1  Bases read: 34841 (x2 for both strands)  Bases in tRNAs: 72  tRNAs predicted: 1  Av. tRNA length: 72  Script CPU time: 0.01 s  Scan CPU time: 0.02 s  Scan speed: 3484.1 Kbp/sec  First pass search(es) ended: Thu Mar 10 03:46:18 PST 2016  Cove Stats:  -----------  Candidate tRNAs read: 1  Cove-confirmed tRNAs: 1  Bases scanned by covels: 86  % seq scanned by covels: 0.1 %  Script CPU time: 0.00 s  Cove CPU time: 0.11 s  Scan speed: 781.8 bp/sec  Cove analysis of tRNAs ended: Thu Mar 10 03:46:18 PST 2016  Summary  --------  Overall scan speed: 497728.6 bp/sec  tRNAs decoding Standard 20 AA: 1  Selenocysteine tRNAs (TCA): 0  Possible suppressor tRNAs (CTA,TTA): 0  tRNAs with undetermined/unknown isotypes: 0  Predicted pseudogenes: 0  -------  Total tRNAs: 1  tRNAs with introns: 0  \|  Isotype / Anticodon Counts:  Ala : 1 AGC: GGC: CGC: 1 TGC:  Gly : 0 ACC: GCC: CCC: TCC:  Pro : 0 AGG: GGG: CGG: TGG:  Thr : 0 AGT: GGT: CGT: TGT:  Val : 0 AAC: GAC: CAC: TAC:  Ser : 0 AGA: GGA: CGA: TGA: ACT: GCT:  Arg : 0 ACG: GCG: CCG: TCG: CCT: TCT:  Leu : 0 AAG: GAG: CAG: TAG: CAA: TAA:  Phe : 0 AAA: GAA:  Asn : 0 ATT: GTT:  Lys : 0 CTT: TTT:  Asp : 0 ATC: GTC:  Glu : 0 CTC: TTC:  His : 0 ATG: GTG:  Gln : 0 CTG: TTG:  Ile : 0 AAT: GAT: TAT:  Met : 0 CAT:  Tyr : 0 ATA: GTA:  Supres: 0 CTA: TTA:  Cys : 0 ACA: GCA:  Trp : 0 CCA:  SelCys: 0 TCA:  **Predicted tRNA Secondary Structures:**  gi\|40217425\|emb\|BX649197.1\|.trna1 (18270-18341) Length: 72 bp  Type: Ala Anticodon: CGC at 33-35 (18302-18304) Score: 75.97  * \| * \| * \| * \| * \| * \| * \|  Seq: GGGCTCGTAGCTCAGTGGAAGAGTGCCTCCTTCGCGAGGAGGAAGcCACGGGTTCAAATCCCGTCGAGTCCA  Str: >>>>>>>..>>>>.......<<<<.>>>>>.......<<<<<.....>>>>>.......<<<<<<<<<<<<.  **Candidate tRNA Predictions in BED format:**  gi\|40217425\|emb\|BX649197.1\| 18269 18341 gi\|40217425\|emb\|BX649197.1\|.tRNA1-AlaCGC 759 + |
