# Supplementary material for: Data on true tRNA diversity among uncultured and bacterial strains
Source: Data Brief. 2016 Apr 26;7:1538–40. doi: 10.1016/j.dib.2016.04.049 (PMC4865659; doi:10.1016/j.dib.2016.04.049)
Supplement: Supplementary file 2 — Supplementary material [file mmc2.docx]

**Table 1**

Results of Analysis of tRNA detected in uncultured bacteria genomes using tRNAScan-SE tool

| **Uncultured bacterium clone zdt-45e5 (AC160099)** |
| --- |
| Sequence tRNA Bounds tRNA Anti Intron Bounds Cove  Name tRNA # Begin End Type Codon Begin End Score  -------- ------ ---- ------ ---- ----- ----- ---- ------   \| gi\|62734802\|gb\|AC160099.1\| 1 2140 2214 Cys GCA 0 0 74.58 \| \| --- \| \| Top of Form  Bottom of Form \| \| gi\|62734802\|gb\|AC160099.1\| 2 8476 8560 Leu TAG 0 0 72.72 \| \| Top of Form  Bottom of Form \| \| gi\|62734802\|gb\|AC160099.1\| 3 11804 11880 Ile GAT 0 0 97.15 \| \| Top of Form  Bottom of Form \| \| gi\|62734802\|gb\|AC160099.1\| 4 12258 12332 Phe GAA 0 0 89.29 \| \| Top of Form  Bottom of Form \| \| gi\|62734802\|gb\|AC160099.1\| 5 12520 12596 Pro TGG 0 0 89.03 \| \| Top of Form  Bottom of Form \| \| gi\|62734802\|gb\|AC160099.1\| 6 12674 12759 Leu CAG 0 0 63.25 \| \| Top of Form  Bottom of Form \| \| gi\|62734802\|gb\|AC160099.1\| 7 12947 13023 Ala CGC 0 0 85.00 \| \| Top of Form  Bottom of Form \| \| gi\|62734802\|gb\|AC160099.1\| 8 13259 13335 Val CAC 0 0 89.71 \| \| Top of Form  Bottom of Form \| \| gi\|62734802\|gb\|AC160099.1\| 9 13453 13529 Pro CGG 0 0 86.73 \| \| Top of Form  Bottom of Form \| \| gi\|62734802\|gb\|AC160099.1\| 10 13704 13781 Pro GGG 0 0 63.78 \| \| Top of Form  Bottom of Form \| \| gi\|62734802\|gb\|AC160099.1\| 11 34094 34007 Leu GAG 0 0 58.87 \| \| Top of Form  Bottom of Form \| \| gi\|62734802\|gb\|AC160099.1\| 12 33822 33746 Val TAC 0 0 91.43 \| \| Top of Form  Bottom of Form \| \| gi\|62734802\|gb\|AC160099.1\| 13 33655 33579 Ala TGC 0 0 91.36 \| \| Top of Form  Bottom of Form \|   **Run Statistics:**  Started: Thu Mar 10 02:35:00 PST 2016  ------------------------------------------------------------  Search Mode: Bacterial  Searching with: tRNAscan + EufindtRNA -> Cove  Covariance model: TRNA2-bact.cm  tRNAscan parameters: Strict  EufindtRNA parameters: Relaxed (Int Cutoff= -36)  ------------------------------------------------------------  First-pass (tRNAscan/EufindtRNA) Stats:  ---------------  Sequences read: 1  Seqs w/at least 1 hit: 1  Bases read: 35743 (x2 for both strands)  Bases in tRNAs: 1157  tRNAs predicted: 14  Av. tRNA length: 82  Script CPU time: 0.01 s  Scan CPU time: 0.02 s  Scan speed: 3574.3 Kbp/sec  First pass search(es) ended: Thu Mar 10 02:35:00 PST 2016  Cove Stats:  -----------  Candidate tRNAs read: 14  Cove-confirmed tRNAs: 13  Bases scanned by covels: 1353  % seq scanned by covels: 1.9 %  Script CPU time: 0.01 s  Cove CPU time: 1.95 s  Scan speed: 693.8 bp/sec  Cove analysis of tRNAs ended: Thu Mar 10 02:35:02 PST 2016  Summary  --------  Overall scan speed: 35922.6 bp/sec  tRNAs decoding Standard 20 AA: 13  Selenocysteine tRNAs (TCA): 0  Possible suppressor tRNAs (CTA,TTA): 0  tRNAs with undetermined/unknown isotypes: 0  Predicted pseudogenes: 0  -------  Total tRNAs: 13  tRNAs with introns: 0  \|  Isotype / Anticodon Counts:  Ala : 2 AGC: GGC: CGC: 1 TGC: 1  Gly : 0 ACC: GCC: CCC: TCC:  Pro : 3 AGG: GGG: 1 CGG: 1 TGG: 1  Thr : 0 AGT: GGT: CGT: TGT:  Val : 2 AAC: GAC: CAC: 1 TAC: 1  Ser : 0 AGA: GGA: CGA: TGA: ACT: GCT:  Arg : 0 ACG: GCG: CCG: TCG: CCT: TCT:  Leu : 3 AAG: GAG: 1 CAG: 1 TAG: 1 CAA: TAA:  Phe : 1 AAA: GAA: 1  Asn : 0 ATT: GTT:  Lys : 0 CTT: TTT:  Asp : 0 ATC: GTC:  Glu : 0 CTC: TTC:  His : 0 ATG: GTG:  Gln : 0 CTG: TTG:  Ile : 1 AAT: GAT: 1 TAT:  Met : 0 CAT:  Tyr : 0 ATA: GTA:  Supres: 0 CTA: TTA:  Cys : 1 ACA: GCA: 1  Trp : 0 CCA:  SelCys: 0 TCA:  **Predicted tRNA Secondary Structures:**  gi\|62734802\|gb\|AC160099.1\|.trna1 (2140-2214) Length: 75 bp  Type: Cys Anticodon: GCA at 33-35 (2172-2174) Score: 74.58  * \| * \| * \| * \| * \| * \| * \|  Seq: GGTCACGTAGCCAAGTGGTAAGGCAGAGGTCTGCAAAACCTTTATcCGGCGGTTCGATTCCGCCCGTGACCTCCA  Str: >>>>>>>..>>>.........<<<.>>>>>.......<<<<<.....>>>>>.......<<<<<<<<<<<<....  gi\|62734802\|gb\|AC160099.1\|.trna2 (8476-8560) Length: 85 bp  Type: Leu Anticodon: TAG at 35-37 (8510-8512) Score: 72.72  * \| * \| * \| * \| * \| * \| * \| * \|  Seq: GCCGGAGTGGTGGAATTGGTaTACACGCATGCCTTAGGAGCATGTGCCGCAAGGCTTGTGAGTTCGAGTCTCGCCTCCGGCACCA  Str: >>>>>>>..>>>...........<<<.>>>>>.......<<<<<.>>>....<<<..>>>>>.......<<<<<<<<<<<<....  gi\|62734802\|gb\|AC160099.1\|.trna3 (11804-11880) Length: 77 bp  Type: Ile Anticodon: GAT at 35-37 (11838-11840) Score: 97.15  * \| * \| * \| * \| * \| * \| * \| *  Seq: GGGCGCGTAGCTCAGTTGGTtAGAGCGCAACACTGATAATGTTGAGGtCCCAAGTTCGATTCTTGGCGCGCCCACCA  Str: >>>>>>>..>>>>.........<<<<.>>>>>.......<<<<<.....>>>>>.......<<<<<<<<<<<<....  gi\|62734802\|gb\|AC160099.1\|.trna4 (12258-12332) Length: 75 bp  Type: Phe Anticodon: GAA at 33-35 (12290-12292) Score: 89.29  * \| * \| * \| * \| * \| * \| * \|  Seq: GCCGCGGTAGCTCAGTGGTAGAGCAGAGGACTGAAAATCCTCGTGtCGGTGGTTCAATTCCGCCCCGCGGCACCA  Str: >>>>>>>..>>>>.......<<<<.>>>>>.......<<<<<.....>>>>>.......<<<<<<<<<<<<....  gi\|62734802\|gb\|AC160099.1\|.trna5 (12520-12596) Length: 77 bp  Type: Pro Anticodon: TGG at 35-37 (12554-12556) Score: 89.03  * \| * \| * \| * \| * \| * \| * \| *  Seq: CGGGGTGTGGCGCAGTTGGCtAGCGCGCGTGGTTTGGGACCATGAGGtCGGAGGTTCAAGTCCTCTCACCCCGACCA  Str: >>>>>>>..>>>>.........<<<<.>>>>>.......<<<<<.....>>>>>.......<<<<<<<<<<<<....  gi\|62734802\|gb\|AC160099.1\|.trna6 (12674-12759) Length: 86 bp  Type: Leu Anticodon: CAG at 35-37 (12708-12710) Score: 63.25  * \| * \| * \| * \| * \| * \| * \| * \| *  Seq: GGGCATGTGGCGGAATTGGCaGACGCGTACGCTTCAGGGGCGTATGGATTTTATCCGTGTGGGTTCAATTCCCGCCATGCCCACCA  Str: >>>>>>>..>>>...........<<<.>>>>>.......<<<<<..>........<..>>>>>.......<<<<<<<<<<<<....  gi\|62734802\|gb\|AC160099.1\|.trna7 (12947-13023) Length: 77 bp  Type: Ala Anticodon: CGC at 35-37 (12981-12983) Score: 85.00  * \| * \| * \| * \| * \| * \| * \| *  Seq: GGGCCTGTAGCTCAGTTGGAtAGAGCGCTACATTCGCATTGTAGAGGtCAGGAGTTCAACTCTCCTCAGGTCCACCA  Str: >>>>>>>..>>>>.........<<<<.>>>>>.......<<<<<.....>>>>>.......<<<<<<<<<<<<....  gi\|62734802\|gb\|AC160099.1\|.trna8 (13259-13335) Length: 77 bp  Type: Val Anticodon: CAC at 35-37 (13293-13295) Score: 89.71  * \| * \| * \| * \| * \| * \| * \| *  Seq: GGGTTTATAGCTCAGCTGGTtAGAGCGTACGGTTCACATCCGTAAGGtCAGAGGTTCAAGTCCTCTTAAACCCACCA  Str: >>>>>>>..>>>>.........<<<<.>>>>>.......<<<<<.....>>>>>.......<<<<<<<<<<<<....  gi\|62734802\|gb\|AC160099.1\|.trna9 (13453-13529) Length: 77 bp  Type: Pro Anticodon: CGG at 35-37 (13487-13489) Score: 86.73  * \| * \| * \| * \| * \| * \| * \| *  Seq: CGGGGATTAGCGCAGTTGGCtAGCGCGCATGCTTCGGGTGCATGAGGtCGTGGGTTCGAGTCCCACATCTCCGACCA  Str: >>>>>>>..>>>>.........<<<<.>>>>>.......<<<<<.....>>>>>.......<<<<<<<<<<<<....  gi\|62734802\|gb\|AC160099.1\|.trna10 (13704-13781) Length: 78 bp  Type: Pro Anticodon: GGG at 36-38 (13739-13741) Score: 63.78  * \| * \| * \| * \| * \| * \| * \| *  Seq: CGGGGTGTAGCATAGTTGGTctAATGCGCAAGGCTGGGGGTCTTGAGAcCGGAGGTTCAAATCCTCTCACCCCGACCA  Str: >>>>>>>..>>>>..........<<<<.>>>>>.......<<<<<.....>>>>>.......<<<<<<<<<<<<....  gi\|62734802\|gb\|AC160099.1\|.trna11 (34094-34007) Length: 88 bp  Type: Leu Anticodon: GAG at 35-37 (34060-34058) Score: 58.87  * \| * \| * \| * \| * \| * \| * \| * \| *  Seq: GTCGGAGTGGTGGAATTGGTaGACACGCAAGCTTGAGGGGCTTGTCCTCATTATGAGGaTGAGGGTTCAAATCCCTCCTTCGACACCA  Str: >>>>>>>..>>>...........<<<.>>>>>.......<<<<<..>>>>...<<<<...>>>>>.......<<<<<<<<<<<<....  gi\|62734802\|gb\|AC160099.1\|.trna12 (33822-33746) Length: 77 bp  Type: Val Anticodon: TAC at 35-37 (33788-33786) Score: 91.43  * \| * \| * \| * \| * \| * \| * \| *  Seq: GGGTCCTTGGCTCAGTTGGTtAGAGCGTCTCGTTTACACCGAGAAGGtCATAGGTTCGACTCCTATAGGACCCACCA  Str: >>>>>>>..>>>>.........<<<<.>>>>>.......<<<<<.....>>>>>.......<<<<<<<<<<<<....  gi\|62734802\|gb\|AC160099.1\|.trna13 (33655-33579) Length: 77 bp  Type: Ala Anticodon: TGC at 35-37 (33621-33619) Score: 91.36  * \| * \| * \| * \| * \| * \| * \| *  Seq: GGGCTCGTAGTTCAGTTGGTtAGAATGCCTGCTTTGCAAGCAGGAGGtCGAGAGTTCGAATCTCTCCGAGTCCACCA  Str: >>>>>>>..>>>>.........<<<<.>>>>>.......<<<<<.....>>>>>.......<<<<<<<<<<<<....  **Candidate tRNA Predictions in BED format:**  gi\|62734802\|gb\|AC160099.1\| 2139 2214 gi\|62734802\|gb\|AC160099.1\|.tRNA1-CysGCA 745 +  gi\|62734802\|gb\|AC160099.1\| 8475 8560 gi\|62734802\|gb\|AC160099.1\|.tRNA2-LeuTAG 727 +  gi\|62734802\|gb\|AC160099.1\| 11803 11880 gi\|62734802\|gb\|AC160099.1\|.tRNA3-IleGAT 971 +  gi\|62734802\|gb\|AC160099.1\| 12257 12332 gi\|62734802\|gb\|AC160099.1\|.tRNA4-PheGAA 892 +  gi\|62734802\|gb\|AC160099.1\| 12519 12596 gi\|62734802\|gb\|AC160099.1\|.tRNA5-ProTGG 890 +  gi\|62734802\|gb\|AC160099.1\| 12673 12759 gi\|62734802\|gb\|AC160099.1\|.tRNA6-LeuCAG 632 +  gi\|62734802\|gb\|AC160099.1\| 12946 13023 gi\|62734802\|gb\|AC160099.1\|.tRNA7-AlaCGC 850 +  gi\|62734802\|gb\|AC160099.1\| 13258 13335 gi\|62734802\|gb\|AC160099.1\|.tRNA8-ValCAC 897 +  gi\|62734802\|gb\|AC160099.1\| 13452 13529 gi\|62734802\|gb\|AC160099.1\|.tRNA9-ProCGG 867 +  gi\|62734802\|gb\|AC160099.1\| 13703 13781 gi\|62734802\|gb\|AC160099.1\|.tRNA10-ProGGG 637 +  gi\|62734802\|gb\|AC160099.1\| 34006 34094 gi\|62734802\|gb\|AC160099.1\|.tRNA11-LeuGAG 588 -  gi\|62734802\|gb\|AC160099.1\| 33745 33822 gi\|62734802\|gb\|AC160099.1\|.tRNA12-ValTAC 914 -  gi\|62734802\|gb\|AC160099.1\| 33578 33655 gi\|62734802\|gb\|AC160099.1\|.tRNA13-AlaTGC 913 - |

| **Uncultured bacterial clone OP32 (FP245540)** |
| --- |
| Sequence tRNA Bounds tRNA Anti Intron Bounds Cove  Name tRNA # Begin End Type Codon Begin End Score  -------- ------ ---- ------ ---- ----- ----- ---- ------   \| gi\|295798128\|emb\|FP245540.1\| 1 3290 3362 Ile GAT 0 0 76.46 \| \| --- \| \| Top of Form  Bottom of Form \| \| gi\|295798128\|emb\|FP245540.1\| 2 3655 3727 Ala TGC 0 0 81.19 \| \| Top of Form  Bottom of Form \| \| gi\|295798128\|emb\|FP245540.1\| 3 10045 10117 Lys CTT 0 0 77.77 \| \| Top of Form  Bottom of Form \| \| gi\|295798128\|emb\|FP245540.1\| 4 20811 20895 Ser TGA 0 0 63.71 \| \| Top of Form  Bottom of Form \|   **Run Statistics:**  Started: Thu Mar 10 02:37:43 PST 2016  ------------------------------------------------------------  Search Mode: Bacterial  Searching with: tRNAscan + EufindtRNA -> Cove  Covariance model: TRNA2-bact.cm  tRNAscan parameters: Strict  EufindtRNA parameters: Relaxed (Int Cutoff= -36)  ------------------------------------------------------------  First-pass (tRNAscan/EufindtRNA) Stats:  ---------------  Sequences read: 1  Seqs w/at least 1 hit: 1  Bases read: 33778 (x2 for both strands)  Bases in tRNAs: 450  tRNAs predicted: 6  Av. tRNA length: 75  Script CPU time: 0.01 s  Scan CPU time: 0.02 s  Scan speed: 3377.8 Kbp/sec  First pass search(es) ended: Thu Mar 10 02:37:43 PST 2016  Cove Stats:  -----------  Candidate tRNAs read: 6  Cove-confirmed tRNAs: 4  Bases scanned by covels: 534  % seq scanned by covels: 0.8 %  Script CPU time: 0.00 s  Cove CPU time: 0.63 s  Scan speed: 847.6 bp/sec  Cove analysis of tRNAs ended: Thu Mar 10 02:37:44 PST 2016  Summary  --------  Overall scan speed: 102357.6 bp/sec  tRNAs decoding Standard 20 AA: 4  Selenocysteine tRNAs (TCA): 0  Possible suppressor tRNAs (CTA,TTA): 0  tRNAs with undetermined/unknown isotypes: 0  Predicted pseudogenes: 0  -------  Total tRNAs: 4  tRNAs with introns: 0  \|  Isotype / Anticodon Counts:  Ala : 1 AGC: GGC: CGC: TGC: 1  Gly : 0 ACC: GCC: CCC: TCC:  Pro : 0 AGG: GGG: CGG: TGG:  Thr : 0 AGT: GGT: CGT: TGT:  Val : 0 AAC: GAC: CAC: TAC:  Ser : 1 AGA: GGA: CGA: TGA: 1 ACT: GCT:  Arg : 0 ACG: GCG: CCG: TCG: CCT: TCT:  Leu : 0 AAG: GAG: CAG: TAG: CAA: TAA:  Phe : 0 AAA: GAA:  Asn : 0 ATT: GTT:  Lys : 1 CTT: 1 TTT:  Asp : 0 ATC: GTC:  Glu : 0 CTC: TTC:  His : 0 ATG: GTG:  Gln : 0 CTG: TTG:  Ile : 1 AAT: GAT: 1 TAT:  Met : 0 CAT:  Tyr : 0 ATA: GTA:  Supres: 0 CTA: TTA:  Cys : 0 ACA: GCA:  Trp : 0 CCA:  SelCys: 0 TCA:  **Predicted tRNA Secondary Structures:**  gi\|295798128\|emb\|FP245540.1\|.trna1 (3290-3362) Length: 73 bp  Type: Ile Anticodon: GAT at 34-36 (3323-3325) Score: 76.46  * \| * \| * \| * \| * \| * \| * \|  Seq: GGGCCCATAGCTCAGTTGGCAGAGCACCGTGCTGATAACGCGGGGGtGACTGGTTCGATCCCAGTTGGGCCCA  Str: >>>>>>>..>>>>........<<<<.>>>>>.......<<<<<.....>>>>>.......<<<<<<<<<<<<.  gi\|295798128\|emb\|FP245540.1\|.trna2 (3655-3727) Length: 73 bp  Type: Ala Anticodon: TGC at 34-36 (3688-3690) Score: 81.19  * \| * \| * \| * \| * \| * \| * \|  Seq: GGGGCAGTAGTTCAGTTGGGAGAACGCCTGCTTTGCAAGCAGGAGGtCCGGAGTTCAACTCTCCGCTGCTCCA  Str: >>>>>>>..>>>>........<<<<.>>>>>.......<<<<<.....>>>>>.......<<<<<<<<<<<<.  gi\|295798128\|emb\|FP245540.1\|.trna3 (10045-10117) Length: 73 bp  Type: Lys Anticodon: CTT at 34-36 (10078-10080) Score: 77.77  * \| * \| * \| * \| * \| * \| * \|  Seq: GGGCCATTAGCTCAATTGGCAGAGCAGGACACTCTTAATGTCAAGGtCGTAGGTTCGACTCCTACATGGCCCA  Str: >>>>>>>..>>>>........<<<<..>>>>.......<<<<......>>>>>.......<<<<<<<<<<<<.  gi\|295798128\|emb\|FP245540.1\|.trna4 (20811-20895) Length: 85 bp  Type: Ser Anticodon: TGA at 35-37 (20845-20847) Score: 63.71  * \| * \| * \| * \| * \| * \| * \| * \|  Seq: GGAGGGGTGGCAGAGCGGTtgAATGCGGCGGTCTTGAAAACCGTTGTGGGGCAACCTACCGTGAGTTCGAATCTCACCCCCTCCG  Str: >>>>>>>..>>>...........<<<.>>>>>.......<<<<<.>>>>>....<<<<<.>>>>>.......<<<<<<<<<<<<.  **Candidate tRNA Predictions in BED format:**  gi\|295798128\|emb\|FP245540.1\| 3289 3362 gi\|295798128\|emb\|FP245540.1\|.tRNA1-IleGAT 764 +  gi\|295798128\|emb\|FP245540.1\| 3654 3727 gi\|295798128\|emb\|FP245540.1\|.tRNA2-AlaTGC 811 +  gi\|295798128\|emb\|FP245540.1\| 10044 10117 gi\|295798128\|emb\|FP245540.1\|.tRNA3-LysCTT 777 +  gi\|295798128\|emb\|FP245540.1\| 20810 20895 gi\|295798128\|emb\|FP245540.1\|.tRNA4-SerTGA 637 + |

| **Uncultured bacterial clone OP33 (FP245539)** |
| --- |
| Sequence tRNA Bounds tRNA Anti Intron Bounds Cove  Name tRNA # Begin End Type Codon Begin End Score  -------- ------ ---- ------ ---- ----- ----- ---- ------   \| gi\|295798098\|emb\|FP245539.1\| 1 23507 23579 Ile GAT 0 0 81.06 \| \| --- \| \| Top of Form  Bottom of Form \| \| gi\|295798098\|emb\|FP245539.1\| 2 23730 23802 Ala TGC 0 0 84.33 \| \| Top of Form  Bottom of Form \|   **Run Statistics:**  Started: Thu Mar 10 02:39:13 PST 2016  ------------------------------------------------------------  Search Mode: Bacterial  Searching with: tRNAscan + EufindtRNA -> Cove  Covariance model: TRNA2-bact.cm  tRNAscan parameters: Strict  EufindtRNA parameters: Relaxed (Int Cutoff= -36)  ------------------------------------------------------------  First-pass (tRNAscan/EufindtRNA) Stats:  ---------------  Sequences read: 1  Seqs w/at least 1 hit: 1  Bases read: 35041 (x2 for both strands)  Bases in tRNAs: 146  tRNAs predicted: 2  Av. tRNA length: 73  Script CPU time: 0.01 s  Scan CPU time: 0.02 s  Scan speed: 3504.1 Kbp/sec  First pass search(es) ended: Thu Mar 10 02:39:13 PST 2016  Cove Stats:  -----------  Candidate tRNAs read: 2  Cove-confirmed tRNAs: 2  Bases scanned by covels: 174  % seq scanned by covels: 0.2 %  Script CPU time: 0.00 s  Cove CPU time: 0.22 s  Scan speed: 790.9 bp/sec  Cove analysis of tRNAs ended: Thu Mar 10 02:39:13 PST 2016  Summary  --------  Overall scan speed: 280328.0 bp/sec  tRNAs decoding Standard 20 AA: 2  Selenocysteine tRNAs (TCA): 0  Possible suppressor tRNAs (CTA,TTA): 0  tRNAs with undetermined/unknown isotypes: 0  Predicted pseudogenes: 0  -------  Total tRNAs: 2  tRNAs with introns: 0  \|  Isotype / Anticodon Counts:  Ala : 1 AGC: GGC: CGC: TGC: 1  Gly : 0 ACC: GCC: CCC: TCC:  Pro : 0 AGG: GGG: CGG: TGG:  Thr : 0 AGT: GGT: CGT: TGT:  Val : 0 AAC: GAC: CAC: TAC:  Ser : 0 AGA: GGA: CGA: TGA: ACT: GCT:  Arg : 0 ACG: GCG: CCG: TCG: CCT: TCT:  Leu : 0 AAG: GAG: CAG: TAG: CAA: TAA:  Phe : 0 AAA: GAA:  Asn : 0 ATT: GTT:  Lys : 0 CTT: TTT:  Asp : 0 ATC: GTC:  Glu : 0 CTC: TTC:  His : 0 ATG: GTG:  Gln : 0 CTG: TTG:  Ile : 1 AAT: GAT: 1 TAT:  Met : 0 CAT:  Tyr : 0 ATA: GTA:  Supres: 0 CTA: TTA:  Cys : 0 ACA: GCA:  Trp : 0 CCA:  SelCys: 0 TCA:  **Predicted tRNA Secondary Structures:**  gi\|295798098\|emb\|FP245539.1\|.trna1 (23507-23579) Length: 73 bp  Type: Ile Anticodon: GAT at 34-36 (23540-23542) Score: 81.06  * \| * \| * \| * \| * \| * \| * \|  Seq: GGGCCCATAGCTCAGTTGGTAGAGCACCGTGTTGATAACGCGGGTGtCAGTGGTTCGAGCCCACTTGGGCCCA  Str: >>>>>>>..>>>>........<<<<.>>>>>.......<<<<<.....>>>>>.......<<<<<<<<<<<<.  gi\|295798098\|emb\|FP245539.1\|.trna2 (23730-23802) Length: 73 bp  Type: Ala Anticodon: TGC at 34-36 (23763-23765) Score: 84.33  * \| * \| * \| * \| * \| * \| * \|  Seq: GGGGGTGTAGCTCAGTTGGGAGAGCGTCTGCTTTGCACGCAGAAGGtCAGGGGTTCAACTCCCCTCACCTCCA  Str: >>>>>>>..>>>>........<<<<.>>>>>.......<<<<<.....>>>>>.......<<<<<<<<<<<<.  **Candidate tRNA Predictions in BED format:**  gi\|295798098\|emb\|FP245539.1\| 23506 23579 gi\|295798098\|emb\|FP245539.1\|.tRNA1-IleGAT 810 +  gi\|295798098\|emb\|FP245539.1\| 23729 23802 gi\|295798098\|emb\|FP245539.1\|.tRNA2-AlaTGC 843 + |

| **Uncultured bacterial clone OP31 (FP245538)** |
| --- |
| Sequence tRNA Bounds tRNA Anti Intron Bounds Cove  Name tRNA # Begin End Type Codon Begin End Score  -------- ------ ---- ------ ---- ----- ----- ---- ------   \| gi\|295798067\|emb\|FP245538.1\| 1 20100 20176 Ile GAT 0 0 85.21 \| \| --- \| \| Top of Form  Bottom of Form \| \| gi\|295798067\|emb\|FP245538.1\| 2 20354 20427 Ala TGC 0 0 82.70 \| \| Top of Form  Bottom of Form \|   **Run Statistics:**  Started: Thu Mar 10 02:48:12 PST 2016  ------------------------------------------------------------  Search Mode: Bacterial  Searching with: tRNAscan + EufindtRNA -> Cove  Covariance model: TRNA2-bact.cm  tRNAscan parameters: Strict  EufindtRNA parameters: Relaxed (Int Cutoff= -36)  ------------------------------------------------------------  First-pass (tRNAscan/EufindtRNA) Stats:  ---------------  Sequences read: 1  Seqs w/at least 1 hit: 1  Bases read: 36635 (x2 for both strands)  Bases in tRNAs: 376  tRNAs predicted: 4  Av. tRNA length: 94  Script CPU time: 0.01 s  Scan CPU time: 0.02 s  Scan speed: 3663.5 Kbp/sec  First pass search(es) ended: Thu Mar 10 02:48:12 PST 2016  Cove Stats:  -----------  Candidate tRNAs read: 4  Cove-confirmed tRNAs: 2  Bases scanned by covels: 432  % seq scanned by covels: 0.6 %  Script CPU time: 0.00 s  Cove CPU time: 0.53 s  Scan speed: 815.1 bp/sec  Cove analysis of tRNAs ended: Thu Mar 10 02:48:13 PST 2016  Summary  --------  Overall scan speed: 130839.3 bp/sec  tRNAs decoding Standard 20 AA: 2  Selenocysteine tRNAs (TCA): 0  Possible suppressor tRNAs (CTA,TTA): 0  tRNAs with undetermined/unknown isotypes: 0  Predicted pseudogenes: 0  -------  Total tRNAs: 2  tRNAs with introns: 0  Isotype / Anticodon Counts:  Ala : 1 AGC: GGC: CGC: TGC: 1  Gly : 0 ACC: GCC: CCC: TCC:  Pro : 0 AGG: GGG: CGG: TGG:  Thr : 0 AGT: GGT: CGT: TGT:  Val : 0 AAC: GAC: CAC: TAC:  Ser : 0 AGA: GGA: CGA: TGA: ACT: GCT:  Arg : 0 ACG: GCG: CCG: TCG: CCT: TCT:  Leu : 0 AAG: GAG: CAG: TAG: CAA: TAA:  Phe : 0 AAA: GAA:  Asn : 0 ATT: GTT:  Lys : 0 CTT: TTT:  Asp : 0 ATC: GTC:  Glu : 0 CTC: TTC:  His : 0 ATG: GTG:  Gln : 0 CTG: TTG:  Ile : 1 AAT: GAT: 1 TAT:  Met : 0 CAT:  Tyr : 0 ATA: GTA:  Supres: 0 CTA: TTA:  Cys : 0 ACA: GCA:  Trp : 0 CCA:  SelCys: 0 TCA:  **Predicted tRNA Secondary Structures:**  gi\|295798067\|emb\|FP245538.1\|.trna1 (20100-20176) Length: 77 bp  Type: Ile Anticodon: GAT at 35-37 (20134-20136) Score: 85.21  * \| * \| * \| * \| * \| * \| * \| *  Seq: GGGCCCGTAGCTCAGATGGCtAGAGCATCGTGCTGATAACGCGGGGGtCAGTGGTTCGATTCCACTCGGGCCCACCA  Str: >>>>>>>..>>>>.........<<<<.>>>>>.......<<<<<.....>>>>>.......<<<<<<<<<<<<....  gi\|295798067\|emb\|FP245538.1\|.trna2 (20354-20427) Length: 74 bp  Type: Ala Anticodon: TGC at 35-37 (20388-20390) Score: 82.70  * \| * \| * \| * \| * \| * \| * \|  Seq: GGGGCTGTAGCTCAGTTtGGGAGAGCGCCTGCTTTGCAAGCAGGAAGtCAGGAGTTCGATCCTCCTCAGCTCCA  Str: >>>>>>>..>>>>.........<<<<.>>>>>.......<<<<<.....>>>>>.......<<<<<<<<<<<<.  **Candidate tRNA Predictions in BED format:**  gi\|295798067\|emb\|FP245538.1\| 20099 20176 gi\|295798067\|emb\|FP245538.1\|.tRNA1-IleGAT 852 +  gi\|295798067\|emb\|FP245538.1\| 20353 20427 gi\|295798067\|emb\|FP245538.1\|.tRNA2-AlaTGC 827 + |

| **Uncultured bacterial clone mtbm116 (FP312985)** |
| --- |
| Sequence tRNA Bounds tRNA Anti Intron Bounds Cove  Name tRNA # Begin End Type Codon Begin End Score  -------- ------ ---- ------ ---- ----- ----- ---- ------   \| gi\|239787676\|emb\|FP312985.1\| 1 5218 5132 Leu CAG 0 0 70.28 \| \| --- \| \| Top of Form  Bottom of Form \|   **Run Statistics:**  Started: Thu Mar 10 03:02:56 PST 2016  ------------------------------------------------------------  Search Mode: Bacterial  Searching with: tRNAscan + EufindtRNA -> Cove  Covariance model: TRNA2-bact.cm  tRNAscan parameters: Strict  EufindtRNA parameters: Relaxed (Int Cutoff= -36)  ------------------------------------------------------------  First-pass (tRNAscan/EufindtRNA) Stats:  ---------------  Sequences read: 1  Seqs w/at least 1 hit: 1  Bases read: 39534 (x2 for both strands)  Bases in tRNAs: 360  tRNAs predicted: 3  Av. tRNA length: 120  Script CPU time: 0.01 s  Scan CPU time: 0.03 s  Scan speed: 2635.6 Kbp/sec  First pass search(es) ended: Thu Mar 10 03:02:56 PST 2016  Cove Stats:  -----------  Candidate tRNAs read: 3  Cove-confirmed tRNAs: 1  Bases scanned by covels: 402  % seq scanned by covels: 0.5 %  Script CPU time: 0.00 s  Cove CPU time: 0.64 s  Scan speed: 628.1 bp/sec  Cove analysis of tRNAs ended: Thu Mar 10 03:02:56 PST 2016  Summary  --------  Overall scan speed: 116276.5 bp/sec  tRNAs decoding Standard 20 AA: 1  Selenocysteine tRNAs (TCA): 0  Possible suppressor tRNAs (CTA,TTA): 0  tRNAs with undetermined/unknown isotypes: 0  Predicted pseudogenes: 0  -------  Total tRNAs: 1  tRNAs with introns: 0  \|  Isotype / Anticodon Counts:  Ala : 0 AGC: GGC: CGC: TGC:  Gly : 0 ACC: GCC: CCC: TCC:  Pro : 0 AGG: GGG: CGG: TGG:  Thr : 0 AGT: GGT: CGT: TGT:  Val : 0 AAC: GAC: CAC: TAC:  Ser : 0 AGA: GGA: CGA: TGA: ACT: GCT:  Arg : 0 ACG: GCG: CCG: TCG: CCT: TCT:  Leu : 1 AAG: GAG: CAG: 1 TAG: CAA: TAA:  Phe : 0 AAA: GAA:  Asn : 0 ATT: GTT:  Lys : 0 CTT: TTT:  Asp : 0 ATC: GTC:  Glu : 0 CTC: TTC:  His : 0 ATG: GTG:  Gln : 0 CTG: TTG:  Ile : 0 AAT: GAT: TAT:  Met : 0 CAT:  Tyr : 0 ATA: GTA:  Supres: 0 CTA: TTA:  Cys : 0 ACA: GCA:  Trp : 0 CCA:  SelCys: 0 TCA:  **Predicted tRNA Secondary Structures:**  gi\|239787676\|emb\|FP312985.1\|.trna1 (5218-5132) Length: 87 bp  Type: Leu Anticodon: CAG at 35-37 (5184-5182) Score: 70.28  * \| * \| * \| * \| * \| * \| * \| * \| *  Seq: GCCCAGGTGGCGGAATTGGTaGACGCGCTAGATTCAGGTTCTAGTGGCTGAAAGGTCGTGGAAGTTCGAGTCTTCTCCTGGGCACCA  Str: >>>>>>>..>>>...........<<<.>>>>>.......<<<<<.>>>>....<<<<..>>>>>.......<<<<<<<<<<<<....  **Candidate tRNA Predictions in BED format:**  gi\|239787676\|emb\|FP312985.1\| 5131 5218 gi\|239787676\|emb\|FP312985.1\|.tRNA1-LeuCAG 702 - |

| **Uncultured bacterial clone mtbe218 (FP312977)** |
| --- |
| Sequence tRNA Bounds tRNA Anti Intron Bounds Cove  Name tRNA # Begin End Type Codon Begin End Score  -------- ------ ---- ------ ---- ----- ----- ---- ------   \| gi\|239787428\|emb\|FP312977.1\| 1 16527 16611 Leu GAG 0 0 61.74 \| \| --- \| \| Top of Form  Bottom of Form \|   **Run Statistics:**  Started: Thu Mar 10 03:08:14 PST 2016  ------------------------------------------------------------  Search Mode: Bacterial  Searching with: tRNAscan + EufindtRNA -> Cove  Covariance model: TRNA2-bact.cm  tRNAscan parameters: Strict  EufindtRNA parameters: Relaxed (Int Cutoff= -36)  ------------------------------------------------------------  First-pass (tRNAscan/EufindtRNA) Stats:  ---------------  Sequences read: 1  Seqs w/at least 1 hit: 1  Bases read: 41140 (x2 for both strands)  Bases in tRNAs: 585  tRNAs predicted: 6  Av. tRNA length: 97  Script CPU time: 0.01 s  Scan CPU time: 0.03 s  Scan speed: 2742.7 Kbp/sec  First pass search(es) ended: Thu Mar 10 03:08:14 PST 2016  Cove Stats:  -----------  Candidate tRNAs read: 6  Cove-confirmed tRNAs: 1  Bases scanned by covels: 669  % seq scanned by covels: 0.8 %  Script CPU time: 0.01 s  Cove CPU time: 0.77 s  Scan speed: 868.8 bp/sec  Cove analysis of tRNAs ended: Thu Mar 10 03:08:14 PST 2016  Summary  --------  Overall scan speed: 100341.5 bp/sec  tRNAs decoding Standard 20 AA: 1  Selenocysteine tRNAs (TCA): 0  Possible suppressor tRNAs (CTA,TTA): 0  tRNAs with undetermined/unknown isotypes: 0  Predicted pseudogenes: 0  -------  Total tRNAs: 1  tRNAs with introns: 0  \|  Isotype / Anticodon Counts:  Ala : 0 AGC: GGC: CGC: TGC:  Gly : 0 ACC: GCC: CCC: TCC:  Pro : 0 AGG: GGG: CGG: TGG:  Thr : 0 AGT: GGT: CGT: TGT:  Val : 0 AAC: GAC: CAC: TAC:  Ser : 0 AGA: GGA: CGA: TGA: ACT: GCT:  Arg : 0 ACG: GCG: CCG: TCG: CCT: TCT:  Leu : 1 AAG: GAG: 1 CAG: TAG: CAA: TAA:  Phe : 0 AAA: GAA:  Asn : 0 ATT: GTT:  Lys : 0 CTT: TTT:  Asp : 0 ATC: GTC:  Glu : 0 CTC: TTC:  His : 0 ATG: GTG:  Gln : 0 CTG: TTG:  Ile : 0 AAT: GAT: TAT:  Met : 0 CAT:  Tyr : 0 ATA: GTA:  Supres: 0 CTA: TTA:  Cys : 0 ACA: GCA:  Trp : 0 CCA:  SelCys: 0 TCA:  **Predicted tRNA Secondary Structures:**  gi\|239787428\|emb\|FP312977.1\|.trna1 (16527-16611) Length: 85 bp  Type: Leu Anticodon: GAG at 35-37 (16561-16563) Score: 61.74  * \| * \| * \| * \| * \| * \| * \| * \|  Seq: GCGGATATGGTGGAATTGGTaGACACGCTGTCTTGAGGGGGCAGTGGCGAGAGCCGTGTCGGTTCAAATCCGACTATCCGCACCA  Str: >>>>>>>..>>>...........<<<.>>>>>.......<<<<<.>>>....<<<..>>>>>.......<<<<<<<<<<<<....  **Candidate tRNA Predictions in BED format:**  gi\|239787428\|emb\|FP312977.1\| 16526 16611 gi\|239787428\|emb\|FP312977.1\|.tRNA1-LeuGAG 617 + |

| **Uncultured bacterial clone magm9502ao02 (FP312975)** |
| --- |
| Sequence tRNA Bounds tRNA Anti Intron Bounds Cove  Name tRNA # Begin End Type Codon Begin End Score  -------- ------ ---- ------ ---- ----- ----- ---- ------   \| gi\|239787359\|emb\|FP312975.1\| 1 17503 17588 Leu GAG 0 0 69.19 \| \| --- \| \| Top of Form  Bottom of Form \|   **Run Statistics:**  Started: Thu Mar 10 03:14:35 PST 2016  ------------------------------------------------------------  Search Mode: Bacterial  Searching with: tRNAscan + EufindtRNA -> Cove  Covariance model: TRNA2-bact.cm  tRNAscan parameters: Strict  EufindtRNA parameters: Relaxed (Int Cutoff= -36)  ------------------------------------------------------------  First-pass (tRNAscan/EufindtRNA) Stats:  ---------------  Sequences read: 1  Seqs w/at least 1 hit: 1  Bases read: 40915 (x2 for both strands)  Bases in tRNAs: 402  tRNAs predicted: 4  Av. tRNA length: 100  Script CPU time: 0.01 s  Scan CPU time: 0.03 s  Scan speed: 2727.7 Kbp/sec  First pass search(es) ended: Thu Mar 10 03:14:35 PST 2016  Cove Stats:  -----------  Candidate tRNAs read: 4  Cove-confirmed tRNAs: 1  Bases scanned by covels: 458  % seq scanned by covels: 0.6 %  Script CPU time: 0.00 s  Cove CPU time: 0.59 s  Scan speed: 776.3 bp/sec  Cove analysis of tRNAs ended: Thu Mar 10 03:14:35 PST 2016  Summary  --------  Overall scan speed: 129888.9 bp/sec  tRNAs decoding Standard 20 AA: 1  Selenocysteine tRNAs (TCA): 0  Possible suppressor tRNAs (CTA,TTA): 0  tRNAs with undetermined/unknown isotypes: 0  Predicted pseudogenes: 0  -------  Total tRNAs: 1  tRNAs with introns: 0  \|  Isotype / Anticodon Counts:  Ala : 0 AGC: GGC: CGC: TGC:  Gly : 0 ACC: GCC: CCC: TCC:  Pro : 0 AGG: GGG: CGG: TGG:  Thr : 0 AGT: GGT: CGT: TGT:  Val : 0 AAC: GAC: CAC: TAC:  Ser : 0 AGA: GGA: CGA: TGA: ACT: GCT:  Arg : 0 ACG: GCG: CCG: TCG: CCT: TCT:  Leu : 1 AAG: GAG: 1 CAG: TAG: CAA: TAA:  Phe : 0 AAA: GAA:  Asn : 0 ATT: GTT:  Lys : 0 CTT: TTT:  Asp : 0 ATC: GTC:  Glu : 0 CTC: TTC:  His : 0 ATG: GTG:  Gln : 0 CTG: TTG:  Ile : 0 AAT: GAT: TAT:  Met : 0 CAT:  Tyr : 0 ATA: GTA:  Supres: 0 CTA: TTA:  Cys : 0 ACA: GCA:  Trp : 0 CCA:  SelCys: 0 TCA:  **Predicted tRNA Secondary Structures:**  gi\|239787359\|emb\|FP312975.1\|.trna1 (17503-17588) Length: 86 bp  Type: Leu Anticodon: GAG at 34-36 (17536-17538) Score: 69.19  * \| * \| * \| * \| * \| * \| * \| * \| *  Seq: GCGGTCGTGGCGGAATGGTaGACGCGCAGCGTTGAGGTCGCTGTGGGGGAAACCCCGTGGAAGTTCGAGTCTTCTCGACCGCACCA  Str: >>>>>>>..>>>..........<<<.>>>>>.......<<<<<.>>>>....<<<<..>>>>>.......<<<<<<<<<<<<....  **Candidate tRNA Predictions in BED format:**  gi\|239787359\|emb\|FP312975.1\| 17502 17588 gi\|239787359\|emb\|FP312975.1\|.tRNA1-LeuGAG 691 + |

| **Uncultured bacterial clone magm9502ae23 (FP312974)** |
| --- |
| Sequence tRNA Bounds tRNA Anti Intron Bounds Cove  Name tRNA # Begin End Type Codon Begin End Score  -------- ------ ---- ------ ---- ----- ----- ---- ------   \| gi\|239787325\|emb\|FP312974.1\| 1 372 445 Gln TTG 0 0 73.53 \| \| --- \| \| Top of Form  Bottom of Form \| \| gi\|239787325\|emb\|FP312974.1\| 2 459 535 Met CAT 0 0 89.86 \| \| Top of Form  Bottom of Form \| \| gi\|239787325\|emb\|FP312974.1\| 3 12028 11955 Gln TTG 0 0 69.62 \| \| Top of Form  Bottom of Form \| \| gi\|239787325\|emb\|FP312974.1\| 4 11941 11865 Met CAT 0 0 89.86 \| \| Top of Form  Bottom of Form \|   **Run Statistics:**  Started: Thu Mar 10 03:18:25 PST 2016  ------------------------------------------------------------  Search Mode: Bacterial  Searching with: tRNAscan + EufindtRNA -> Cove  Covariance model: TRNA2-bact.cm  tRNAscan parameters: Strict  EufindtRNA parameters: Relaxed (Int Cutoff= -36)  ------------------------------------------------------------  First-pass (tRNAscan/EufindtRNA) Stats:  ---------------  Sequences read: 1  Seqs w/at least 1 hit: 1  Bases read: 39577 (x2 for both strands)  Bases in tRNAs: 366  tRNAs predicted: 5  Av. tRNA length: 73  Script CPU time: 0.01 s  Scan CPU time: 0.03 s  Scan speed: 2638.5 Kbp/sec  First pass search(es) ended: Thu Mar 10 03:18:25 PST 2016  Cove Stats:  -----------  Candidate tRNAs read: 5  Cove-confirmed tRNAs: 4  Bases scanned by covels: 436  % seq scanned by covels: 0.6 %  Script CPU time: 0.00 s  Cove CPU time: 0.53 s  Scan speed: 822.6 bp/sec  Cove analysis of tRNAs ended: Thu Mar 10 03:18:25 PST 2016  Summary  --------  Overall scan speed: 138866.7 bp/sec  tRNAs decoding Standard 20 AA: 4  Selenocysteine tRNAs (TCA): 0  Possible suppressor tRNAs (CTA,TTA): 0  tRNAs with undetermined/unknown isotypes: 0  Predicted pseudogenes: 0  -------  Total tRNAs: 4  tRNAs with introns: 0  \|  Isotype / Anticodon Counts:  Ala : 0 AGC: GGC: CGC: TGC:  Gly : 0 ACC: GCC: CCC: TCC:  Pro : 0 AGG: GGG: CGG: TGG:  Thr : 0 AGT: GGT: CGT: TGT:  Val : 0 AAC: GAC: CAC: TAC:  Ser : 0 AGA: GGA: CGA: TGA: ACT: GCT:  Arg : 0 ACG: GCG: CCG: TCG: CCT: TCT:  Leu : 0 AAG: GAG: CAG: TAG: CAA: TAA:  Phe : 0 AAA: GAA:  Asn : 0 ATT: GTT:  Lys : 0 CTT: TTT:  Asp : 0 ATC: GTC:  Glu : 0 CTC: TTC:  His : 0 ATG: GTG:  Gln : 2 CTG: TTG: 2  Ile : 0 AAT: GAT: TAT:  Met : 2 CAT: 2  Tyr : 0 ATA: GTA:  Supres: 0 CTA: TTA:  Cys : 0 ACA: GCA:  Trp : 0 CCA:  SelCys: 0 TCA:  **Predicted tRNA Secondary Structures:**  gi\|239787325\|emb\|FP312974.1\|.trna1 (372-445) Length: 74 bp  Type: Gln Anticodon: TTG at 33-35 (404-406) Score: 73.53  * \| * \| * \| * \| * \| * \| * \|  Seq: TGGGGTATAGCCAAGTGGTAAGGCATCGGTTTTTGGTACCGACAtCCTAGGTTCGAATCCTAGTACCCCAGCCA  Str: >>>>>>>..>>>.........<<<.>>>>>.......<<<<<....>>>>>.......<<<<<<<<<<<<....  gi\|239787325\|emb\|FP312974.1\|.trna2 (459-535) Length: 77 bp  Type: Met Anticodon: CAT at 35-37 (493-495) Score: 89.86  * \| * \| * \| * \| * \| * \| * \| *  Seq: GGCGGAGTAGCTCAGCTGGTtAGAGCGACGGAATCATAATCCGCAGGtCGGGGGTTCGAATCCCTCCTCCGCTACCA  Str: >>>>>>>..>>>>.........<<<<..>>>>.......<<<<......>>>>>.......<<<<<<<<<<<<....  gi\|239787325\|emb\|FP312974.1\|.trna3 (12028-11955) Length: 74 bp  Type: Gln Anticodon: TTG at 33-35 (11996-11994) Score: 69.62  * \| * \| * \| * \| * \| * \| * \|  Seq: GGGGGTATAGCCAAGTGGTAAGGCATCGGTTTTTGGTACCGACAtCCTAGGTTCGAATCCTAGTACCCCAGCCA  Str: .>>>>>>..>>>.........<<<.>>>>>.......<<<<<....>>>>>.......<<<<<<<<<<<.....  gi\|239787325\|emb\|FP312974.1\|.trna4 (11941-11865) Length: 77 bp  Type: Met Anticodon: CAT at 35-37 (11907-11905) Score: 89.86  * \| * \| * \| * \| * \| * \| * \| *  Seq: GGCGGAGTAGCTCAGCTGGTtAGAGCGACGGAATCATAATCCGCAGGtCGGGGGTTCGAATCCCTCCTCCGCTACCA  Str: >>>>>>>..>>>>.........<<<<..>>>>.......<<<<......>>>>>.......<<<<<<<<<<<<....  **Candidate tRNA Predictions in BED format:**  gi\|239787325\|emb\|FP312974.1\| 371 445 gi\|239787325\|emb\|FP312974.1\|.tRNA1-GlnTTG 735 +  gi\|239787325\|emb\|FP312974.1\| 458 535 gi\|239787325\|emb\|FP312974.1\|.tRNA2-MetCAT 898 +  gi\|239787325\|emb\|FP312974.1\| 11954 12028 gi\|239787325\|emb\|FP312974.1\|.tRNA3-GlnTTG 696 -  gi\|239787325\|emb\|FP312974.1\| 11864 11941 gi\|239787325\|emb\|FP312974.1\|.tRNA4-MetCAT 898 - |

| **Uncultured bacterial clone mtbe94 (FP312972)** |
| --- |
| Sequence tRNA Bounds tRNA Anti Intron Bounds Cove  Name tRNA # Begin End Type Codon Begin End Score  -------- ------ ---- ------ ---- ----- ----- ---- ------   \| gi\|239787267\|emb\|FP312972.1\| 1 15699 15774 Glu CTC 0 0 58.09 \| \| --- \| \| Top of Form  Bottom of Form \|   **Run Statistics:**  Started: Thu Mar 10 03:20:19 PST 2016  ------------------------------------------------------------  Search Mode: Bacterial  Searching with: tRNAscan + EufindtRNA -> Cove  Covariance model: TRNA2-bact.cm  tRNAscan parameters: Strict  EufindtRNA parameters: Relaxed (Int Cutoff= -36)  ------------------------------------------------------------  First-pass (tRNAscan/EufindtRNA) Stats:  ---------------  Sequences read: 1  Seqs w/at least 1 hit: 1  Bases read: 34505 (x2 for both strands)  Bases in tRNAs: 311  tRNAs predicted: 3  Av. tRNA length: 103  Script CPU time: 0.00 s  Scan CPU time: 0.03 s  Scan speed: 2300.3 Kbp/sec  First pass search(es) ended: Thu Mar 10 03:20:19 PST 2016  Cove Stats:  -----------  Candidate tRNAs read: 3  Cove-confirmed tRNAs: 1  Bases scanned by covels: 353  % seq scanned by covels: 0.5 %  Script CPU time: 0.01 s  Cove CPU time: 0.43 s  Scan speed: 820.9 bp/sec  Cove analysis of tRNAs ended: Thu Mar 10 03:20:20 PST 2016  Summary  --------  Overall scan speed: 146829.8 bp/sec  tRNAs decoding Standard 20 AA: 1  Selenocysteine tRNAs (TCA): 0  Possible suppressor tRNAs (CTA,TTA): 0  tRNAs with undetermined/unknown isotypes: 0  Predicted pseudogenes: 0  -------  Total tRNAs: 1  tRNAs with introns: 0  \|  Isotype / Anticodon Counts:  Ala : 0 AGC: GGC: CGC: TGC:  Gly : 0 ACC: GCC: CCC: TCC:  Pro : 0 AGG: GGG: CGG: TGG:  Thr : 0 AGT: GGT: CGT: TGT:  Val : 0 AAC: GAC: CAC: TAC:  Ser : 0 AGA: GGA: CGA: TGA: ACT: GCT:  Arg : 0 ACG: GCG: CCG: TCG: CCT: TCT:  Leu : 0 AAG: GAG: CAG: TAG: CAA: TAA:  Phe : 0 AAA: GAA:  Asn : 0 ATT: GTT:  Lys : 0 CTT: TTT:  Asp : 0 ATC: GTC:  Glu : 1 CTC: 1 TTC:  His : 0 ATG: GTG:  Gln : 0 CTG: TTG:  Ile : 0 AAT: GAT: TAT:  Met : 0 CAT:  Tyr : 0 ATA: GTA:  Supres: 0 CTA: TTA:  Cys : 0 ACA: GCA:  Trp : 0 CCA:  SelCys: 0 TCA:  **Predicted tRNA Secondary Structures:**  gi\|239787267\|emb\|FP312972.1\|.trna1 (15699-15774) Length: 76 bp  Type: Glu Anticodon: CTC at 35-37 (15733-15735) Score: 58.09  * \| * \| * \| * \| * \| * \| * \| *  Seq: GTCCCCATCGTCTAGTGGCctAGGACGCTGGCCTCTCACGCCGGTAACAGGGGTTCGACTCCCCTTGGGGACGCCA  Str: >>>>>>>..>>>>.........<<<<.>>>>>.......<<<<<....>>>>>.......<<<<<<<<<<<<....  **Candidate tRNA Predictions in BED format:**  gi\|239787267\|emb\|FP312972.1\| 15698 15774 gi\|239787267\|emb\|FP312972.1\|.tRNA1-GluCTC 580 + |

| ***Oscillibacter* sp. ER4 contig_75 (NZ_JPJG01000067)** |
| --- |
| Sequence tRNA Bounds tRNA Anti Intron Bounds Cove  Name tRNA # Begin End Type Codon Begin End Score  -------- ------ ---- ------ ---- ----- ----- ---- ------   \| gi\|696600845\|ref\|NZ_JPJG01000067.1\| 1 1560 1484 Ile GAT 0 0 96.25 \| \| --- \| \| Top of Form  Bottom of Form \|   **Run Statistics:**  Started: Thu Mar 10 03:22:06 PST 2016  ------------------------------------------------------------  Search Mode: Bacterial  Searching with: tRNAscan + EufindtRNA -> Cove  Covariance model: TRNA2-bact.cm  tRNAscan parameters: Strict  EufindtRNA parameters: Relaxed (Int Cutoff= -36)  ------------------------------------------------------------  First-pass (tRNAscan/EufindtRNA) Stats:  ---------------  Sequences read: 1  Seqs w/at least 1 hit: 1  Bases read: 1560 (x2 for both strands)  Bases in tRNAs: 74  tRNAs predicted: 1  Av. tRNA length: 74  Script CPU time: 0.00 s  Scan CPU time: 0.00 s  Scan speed: 3120.0 Kbp/sec  First pass search(es) ended: Thu Mar 10 03:22:06 PST 2016  Cove Stats:  -----------  Candidate tRNAs read: 1  Cove-confirmed tRNAs: 1  Bases scanned by covels: 81  % seq scanned by covels: 2.6 %  Script CPU time: 0.01 s  Cove CPU time: 0.11 s  Scan speed: 736.4 bp/sec  Cove analysis of tRNAs ended: Thu Mar 10 03:22:07 PST 2016  Summary  --------  Overall scan speed: 26000.0 bp/sec  tRNAs decoding Standard 20 AA: 1  Selenocysteine tRNAs (TCA): 0  Possible suppressor tRNAs (CTA,TTA): 0  tRNAs with undetermined/unknown isotypes: 0  Predicted pseudogenes: 0  -------  Total tRNAs: 1  tRNAs with introns: 0  \|  Isotype / Anticodon Counts:  Ala : 0 AGC: GGC: CGC: TGC:  Gly : 0 ACC: GCC: CCC: TCC:  Pro : 0 AGG: GGG: CGG: TGG:  Thr : 0 AGT: GGT: CGT: TGT:  Val : 0 AAC: GAC: CAC: TAC:  Ser : 0 AGA: GGA: CGA: TGA: ACT: GCT:  Arg : 0 ACG: GCG: CCG: TCG: CCT: TCT:  Leu : 0 AAG: GAG: CAG: TAG: CAA: TAA:  Phe : 0 AAA: GAA:  Asn : 0 ATT: GTT:  Lys : 0 CTT: TTT:  Asp : 0 ATC: GTC:  Glu : 0 CTC: TTC:  His : 0 ATG: GTG:  Gln : 0 CTG: TTG:  Ile : 1 AAT: GAT: 1 TAT:  Met : 0 CAT:  Tyr : 0 ATA: GTA:  Supres: 0 CTA: TTA:  Cys : 0 ACA: GCA:  Trp : 0 CCA:  SelCys: 0 TCA:  **Predicted tRNA Secondary Structures:**  gi\|696600845\|ref\|NZ_JPJG01000067.1\|.trna1 (1560-1484) Length: 77 bp  Type: Ile Anticodon: GAT at 35-37 (1526-1524) Score: 96.25  * \| * \| * \| * \| * \| * \| * \| *  Seq: GGGCCCGTAGCTCAGCTGGCtAGAGCGTACGACTGATAATCGTAAGGtCGGTGGTTCGAGCCCACTCGGGCCCACCA  Str: >>>>>>>..>>>>.........<<<<.>>>>>.......<<<<<.....>>>>>.......<<<<<<<<<<<<....  **Candidate tRNA Predictions in BED format:**  gi\|696600845\|ref\|NZ_JPJG01000067.1\| 1483 1560 gi\|696600845\|ref\|NZ_JPJG01000067.1\|.tRNA1-IleGAT 962 - |
