# Supplementary material for: Data on true tRNA diversity among uncultured and bacterial strains
Source: Data Brief. 2016 Apr 26;7:1538–40. doi: 10.1016/j.dib.2016.04.049 (PMC4865659; doi:10.1016/j.dib.2016.04.049)
Supplement: Supplementary file 3 — Supplementary material [file mmc3.docx]

| Uncultured bacterium clone zdt-45e5 (AC160099) | |
| --- | --- |
| 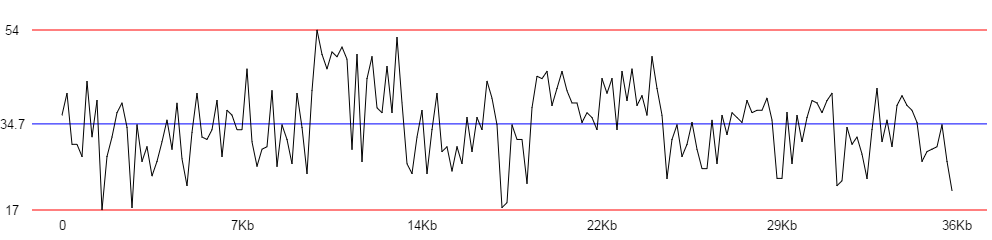 | |
| 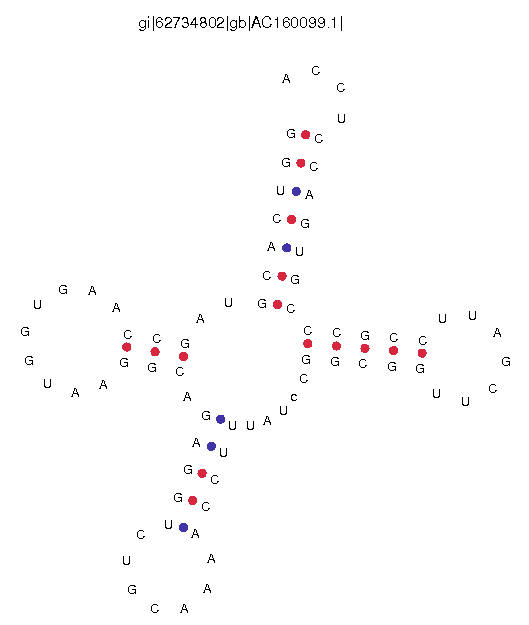 | 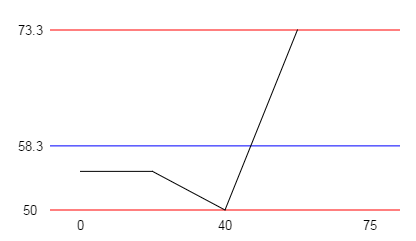 |
| 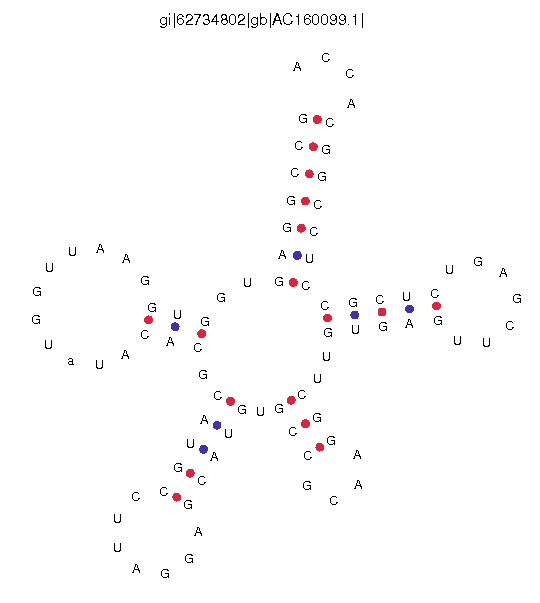 | 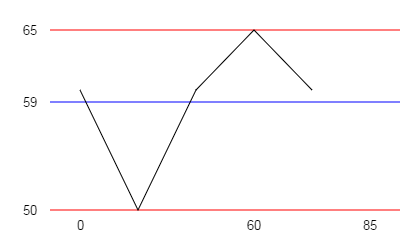 |
| 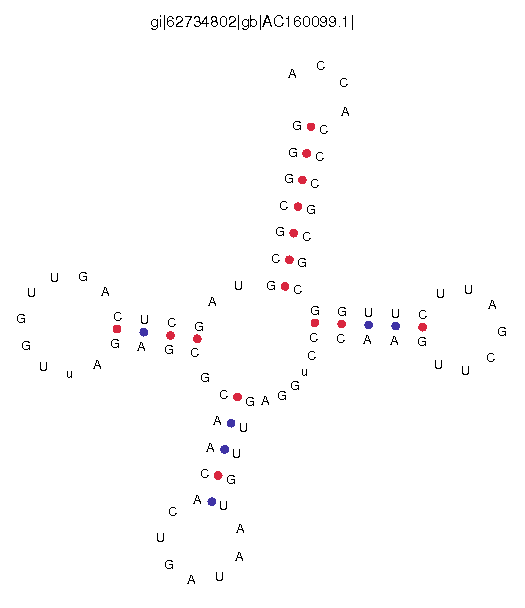 | 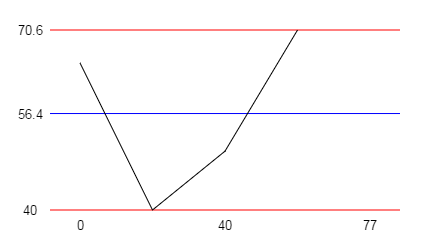 |
| 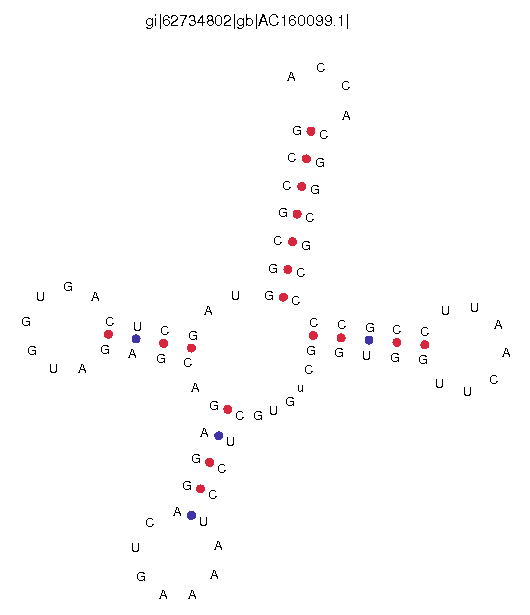 | 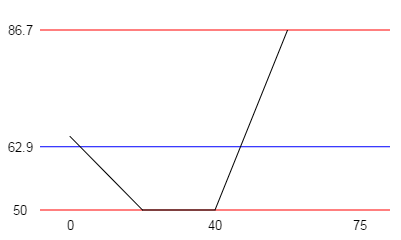 |
| 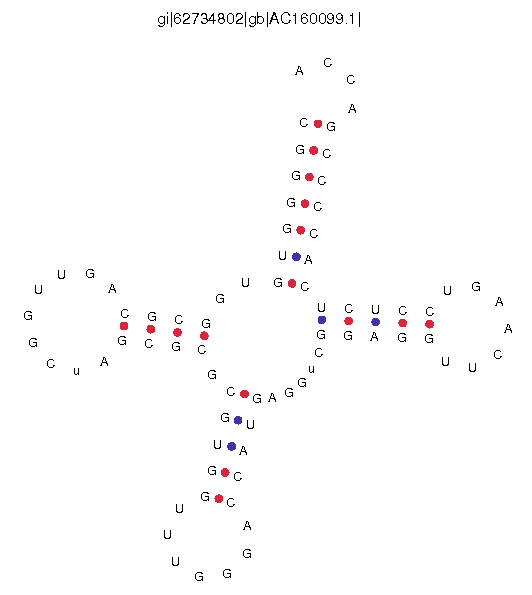 | 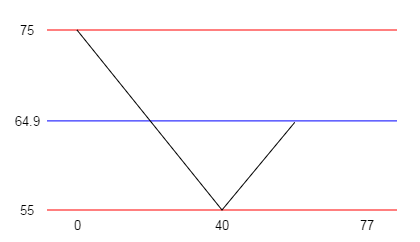 |
| 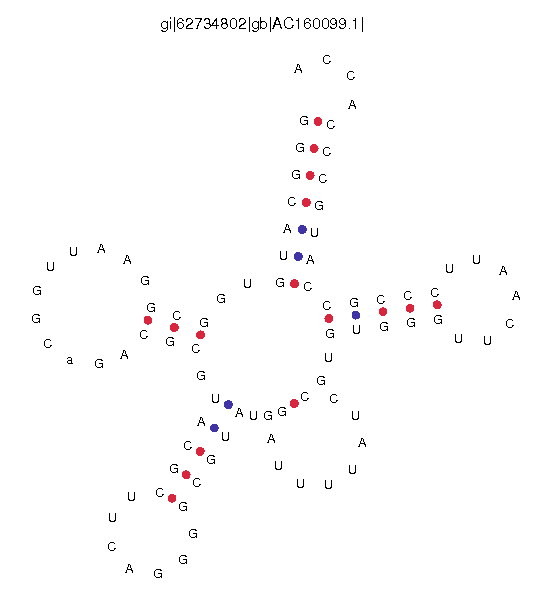 | 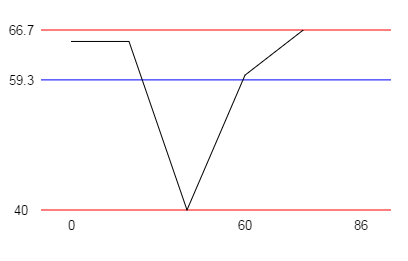 |
| 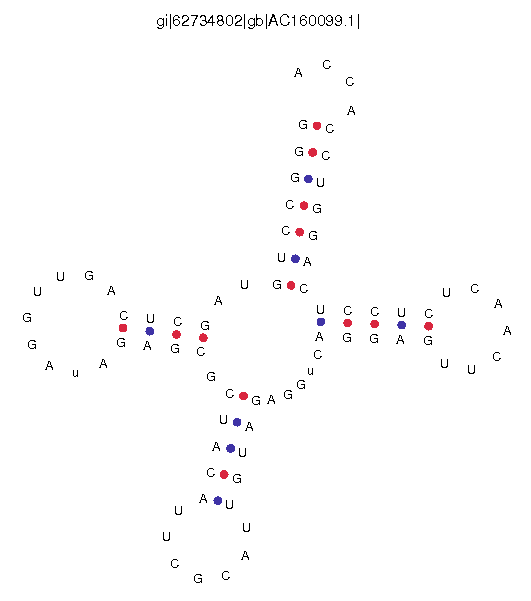 | 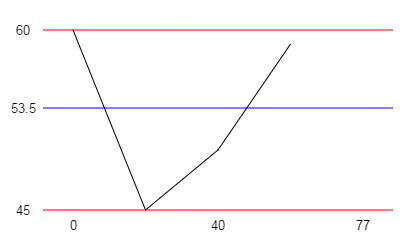 |
| 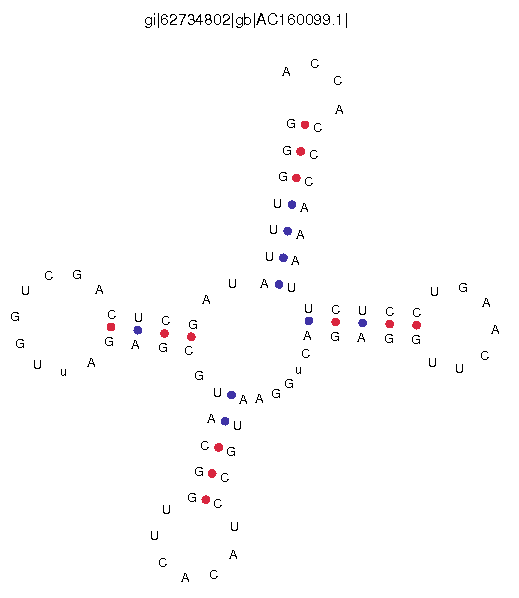 | 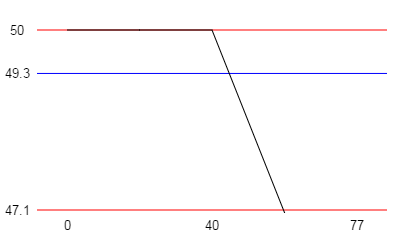 |
| 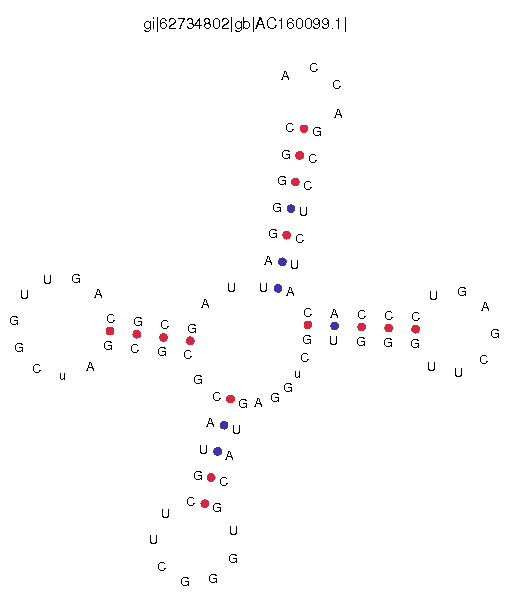 | 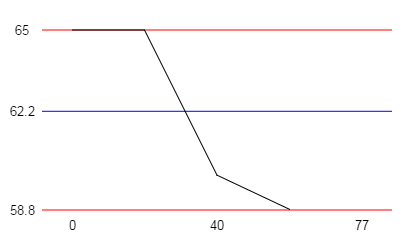 |
| 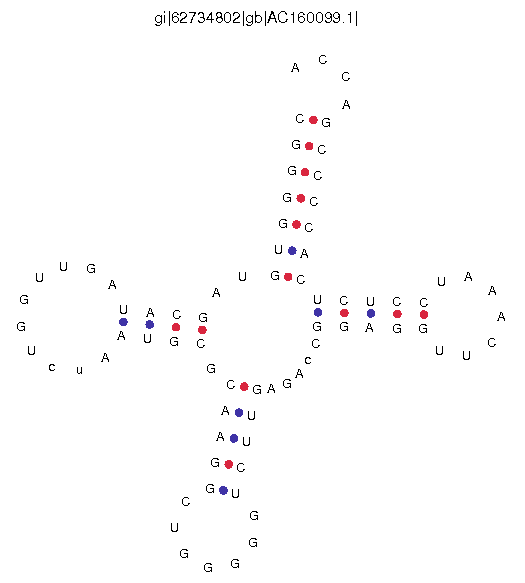 | 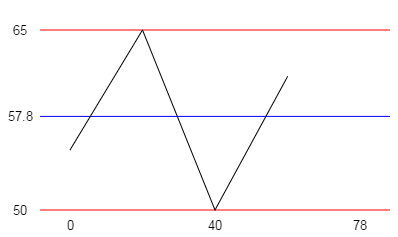 |
| 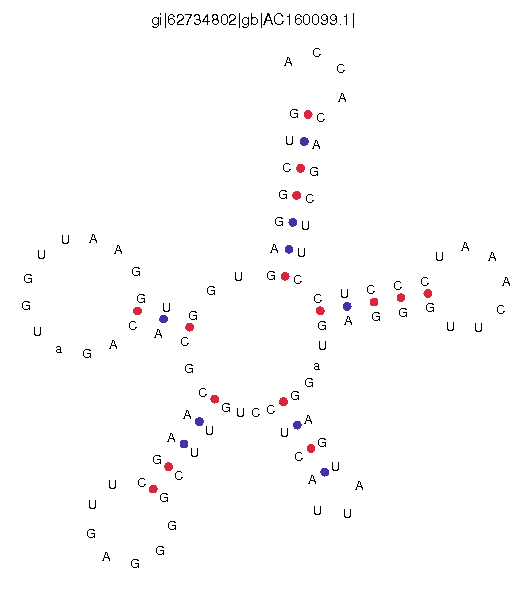 | 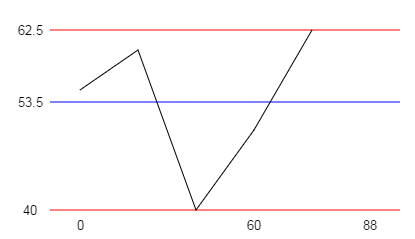 |
| 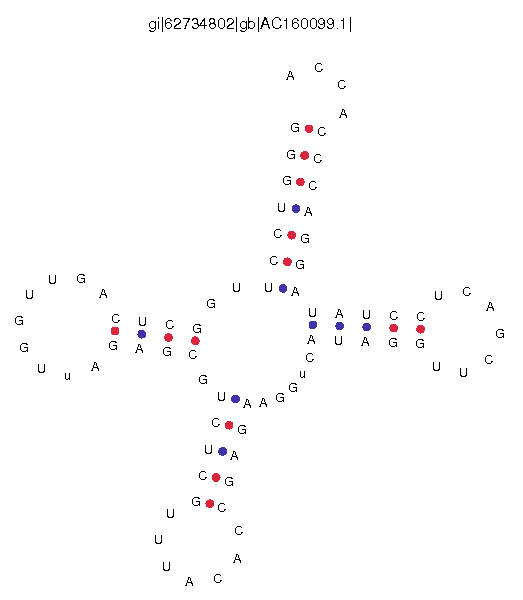 | 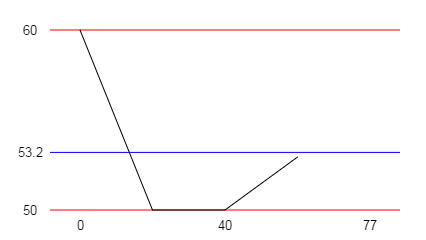 |
| 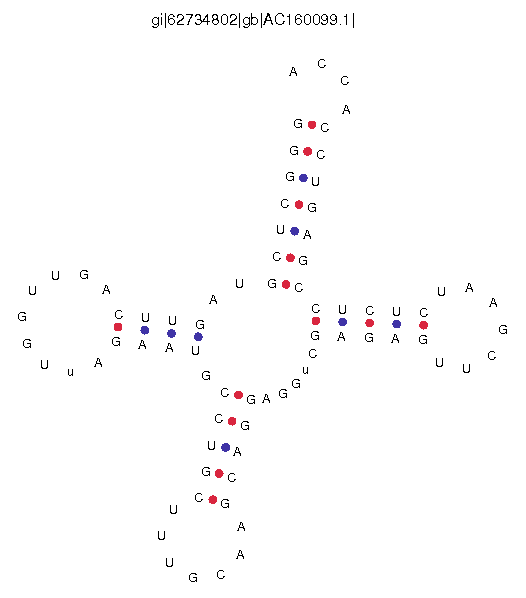 | 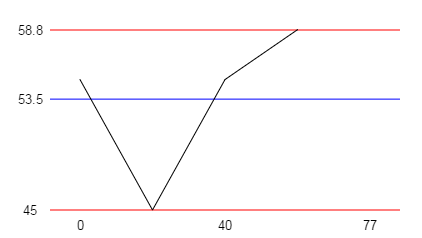 |
| Uncultured archaeon clone 0418F12 (BX649197) | |
| 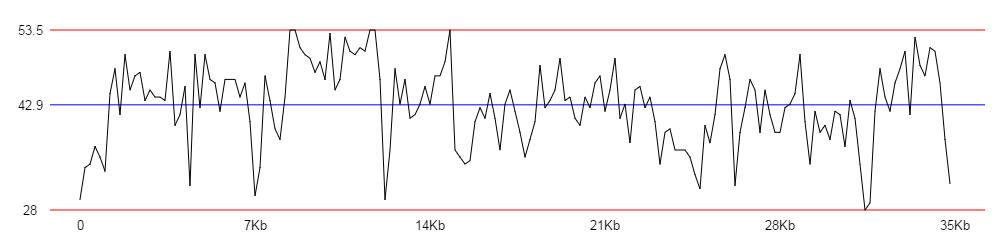 | |
| 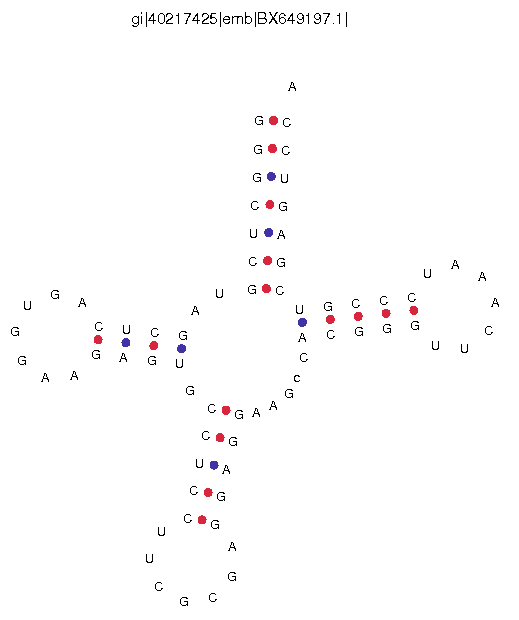 | 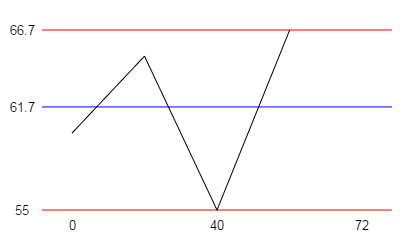 |
| Uncultured archaeon clone fos0128g3+03e1 (CR937008) | |
| 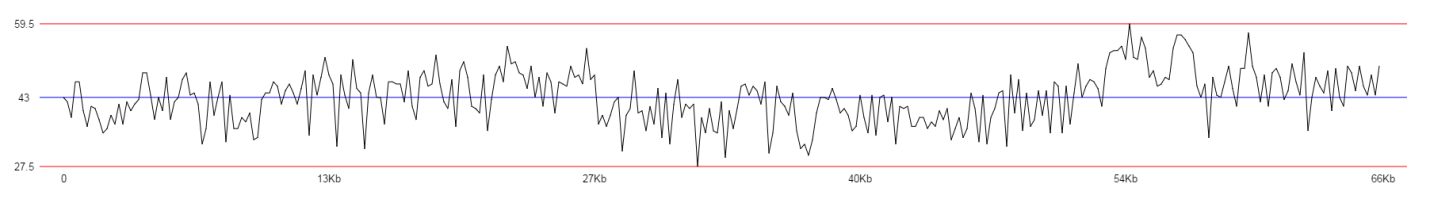 | |
| 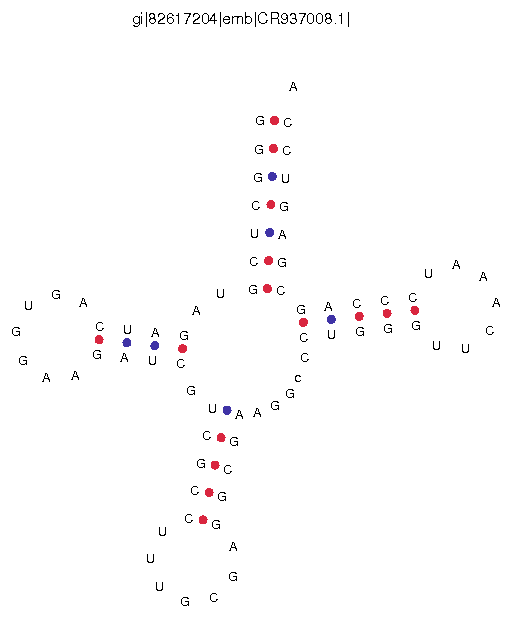 | 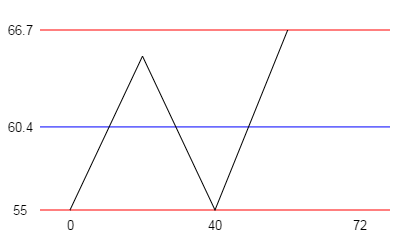 |
| 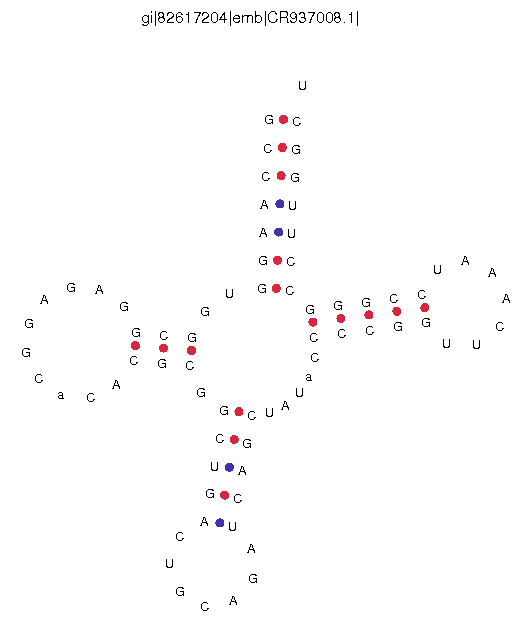 | 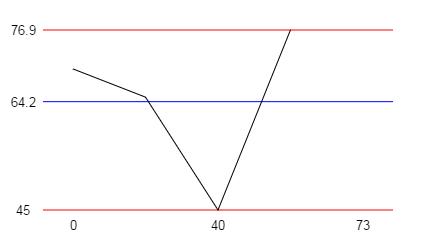 |
| 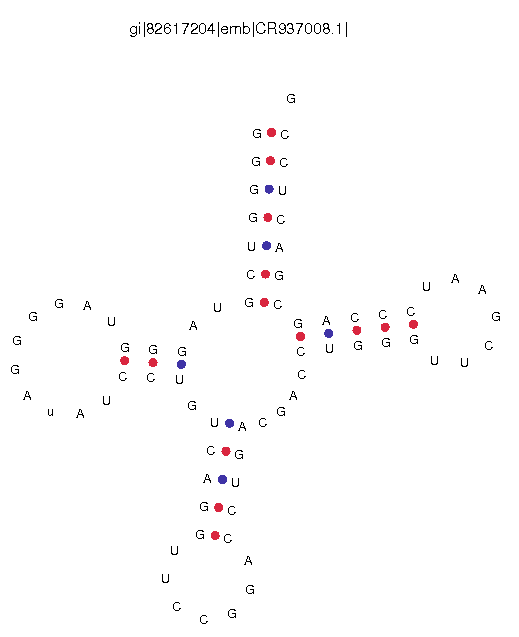 | 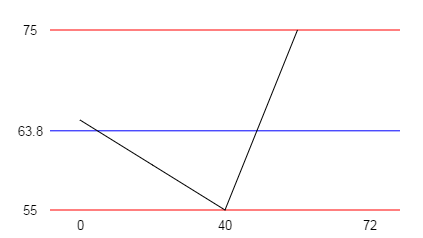 |
| Uncultured bacterial clone OP31 (FP245538) | |
| 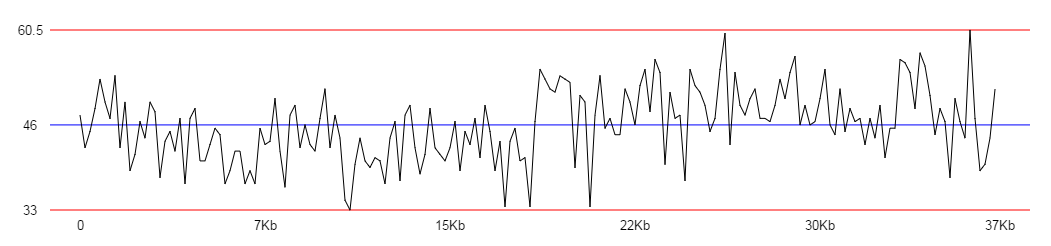 | |
| 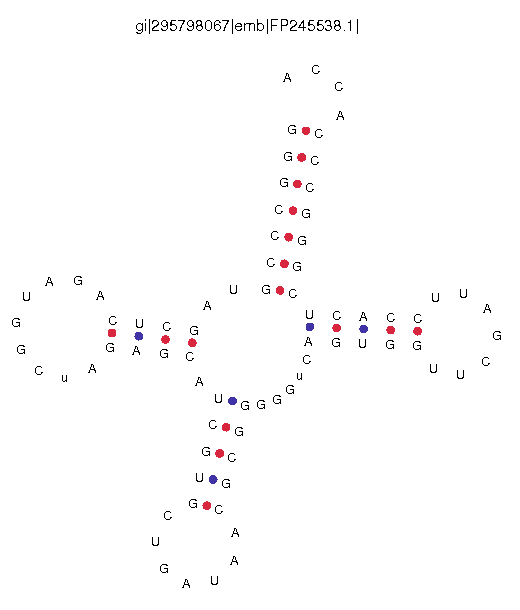 | 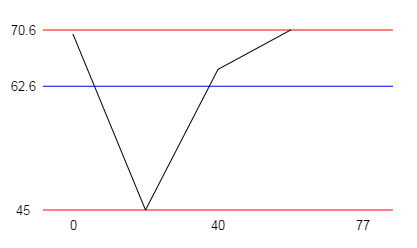 |
| 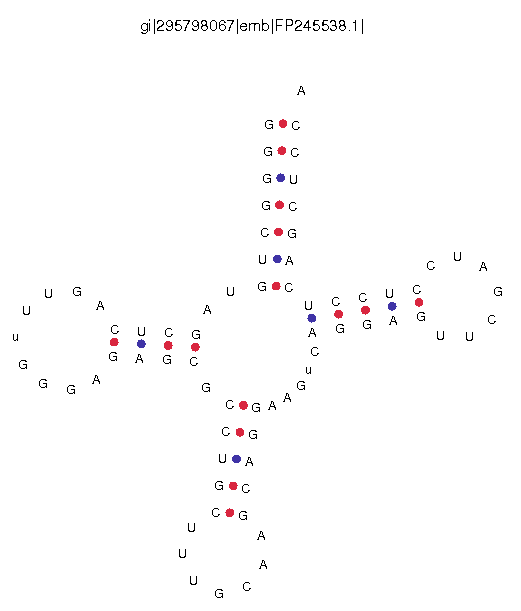 | 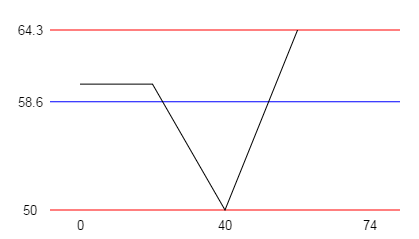 |
| Uncultured bacterial clone OP33 (FP245539) | |
| 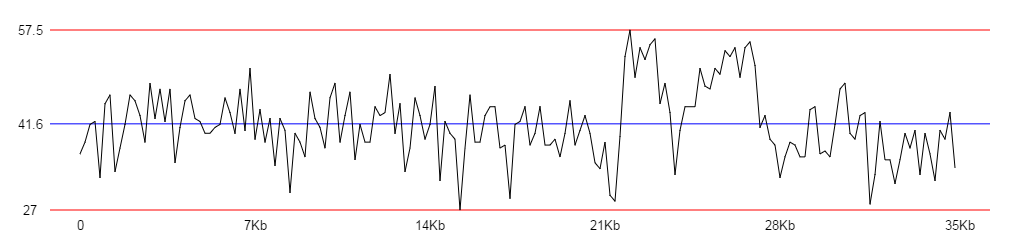 | |
| 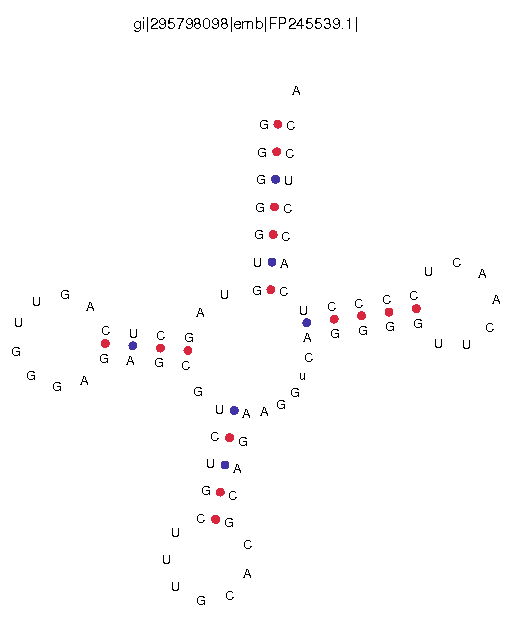 | 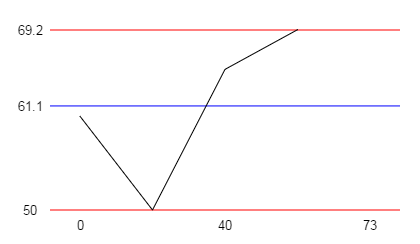 |
| 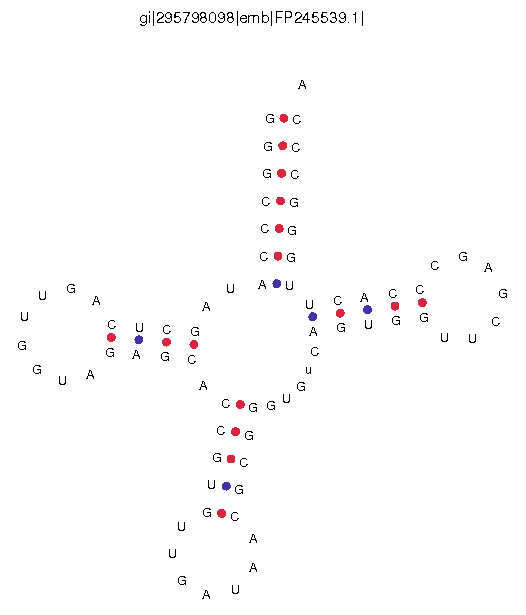 | 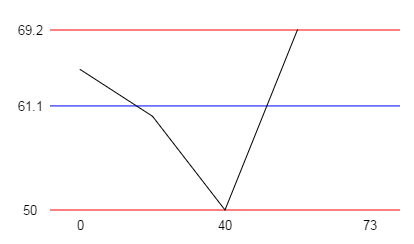 |
| Uncultured bacterial clone OP32 (FP245540) | |
| 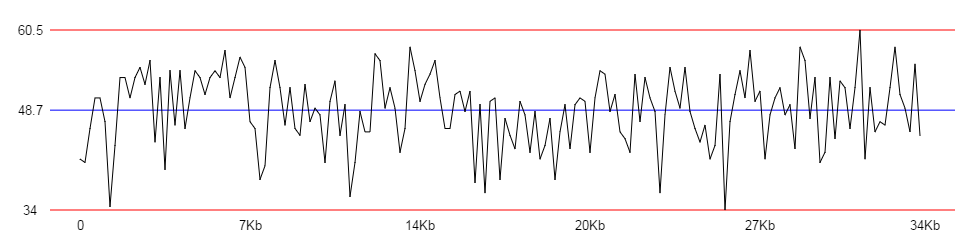 | |
| 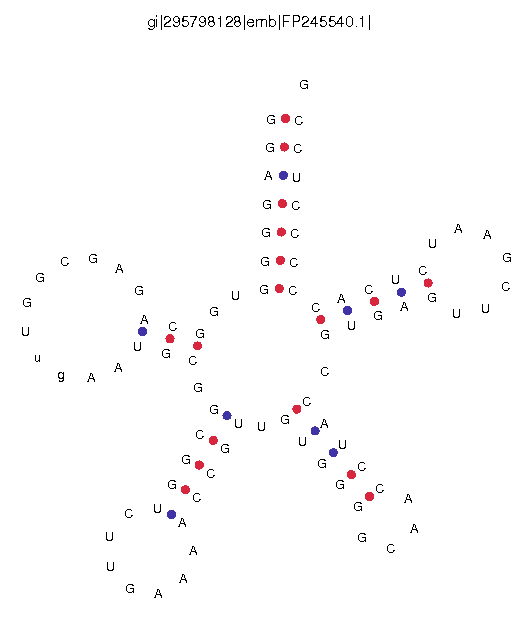 | 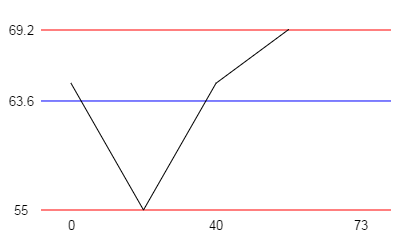 |
| 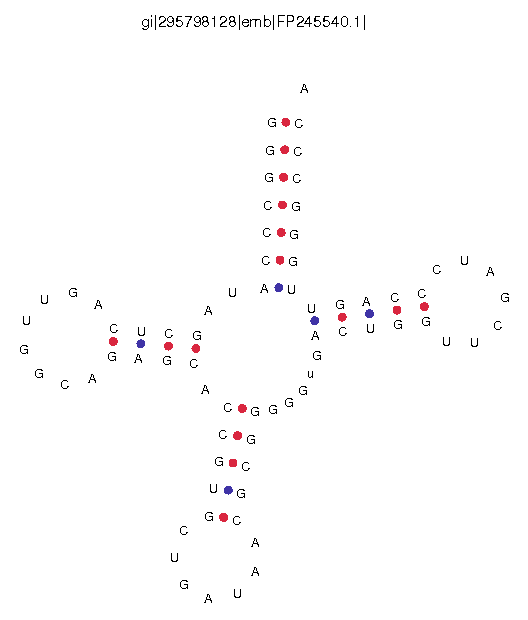 | 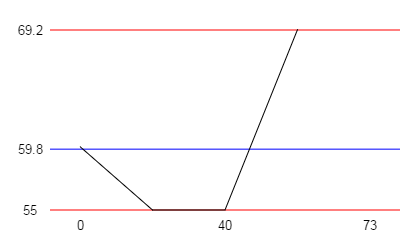 |
| 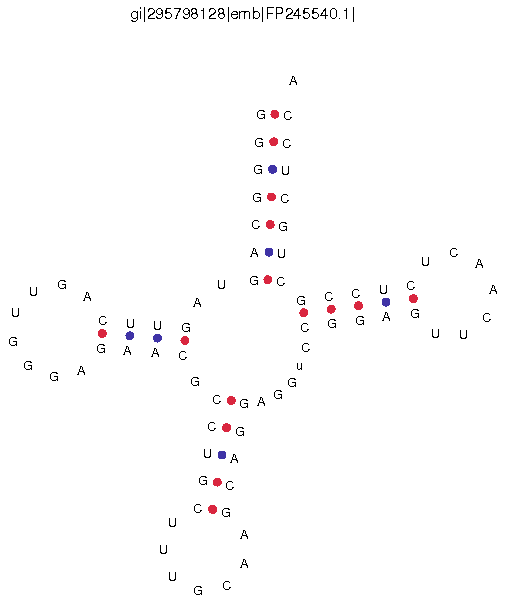 | 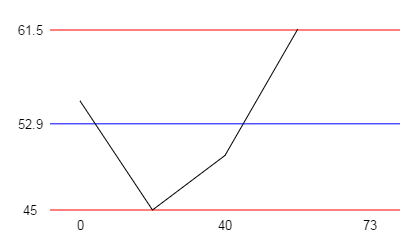 |
| 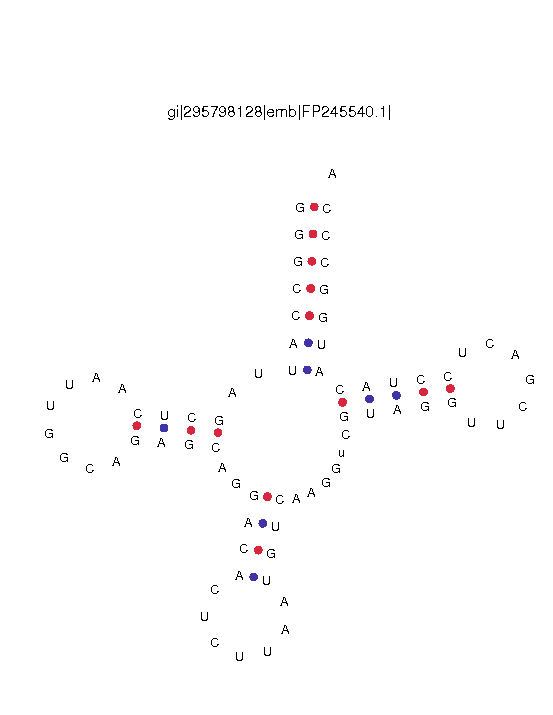 | 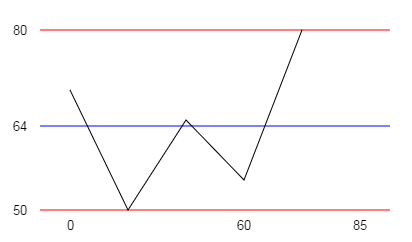 |
| Uncultured bacterial clone mtbe94 (FP312972) | |
| 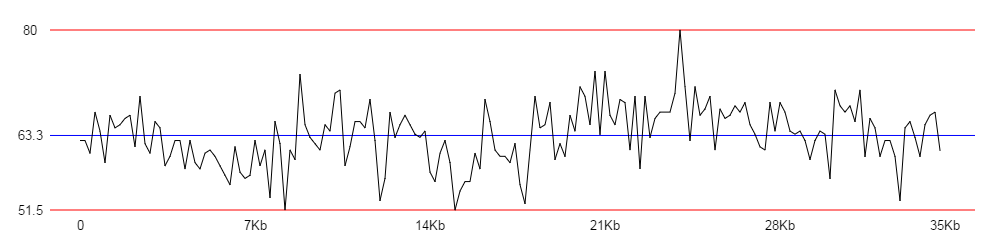 | |
| 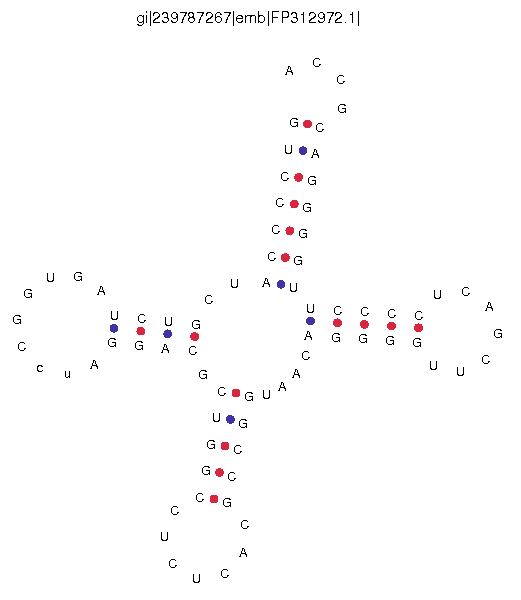 | 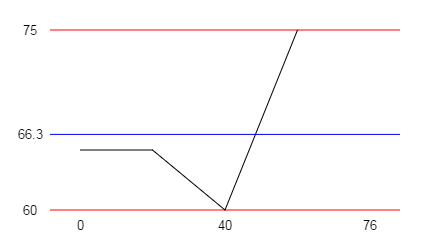 |
| Uncultured bacterial clone magm9502ae23 (FP312974) | |
| 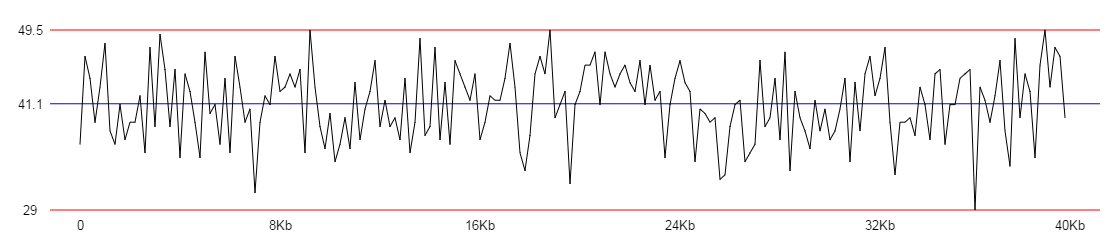 | |
| 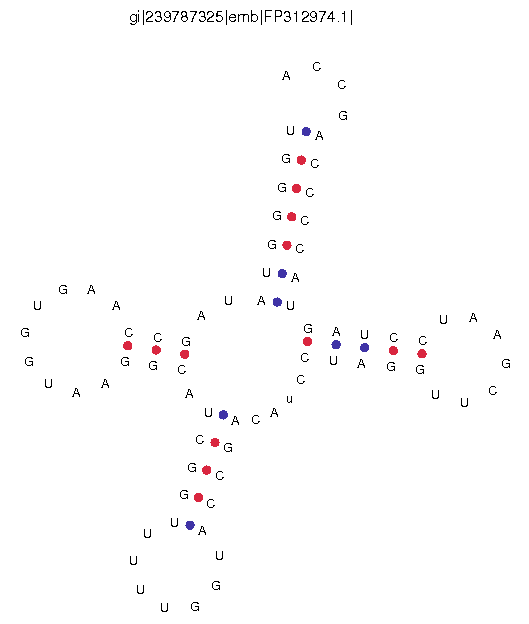 | 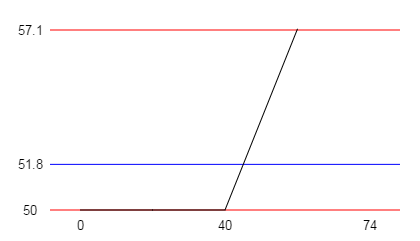 |
| 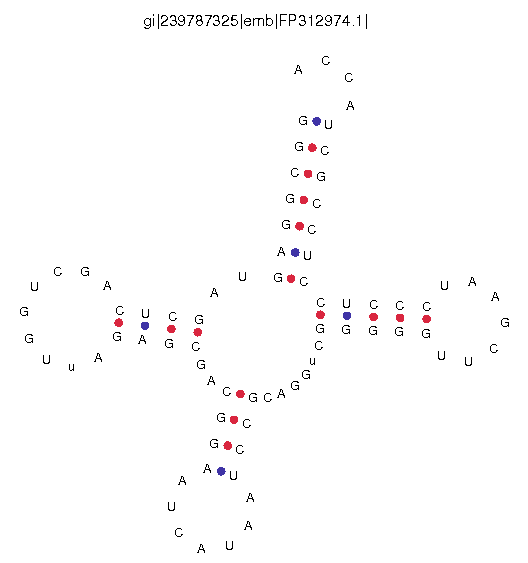 | 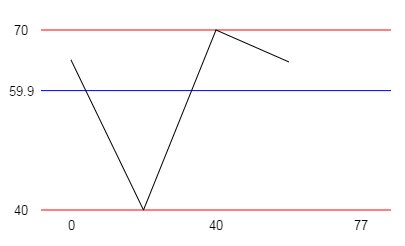 |
| 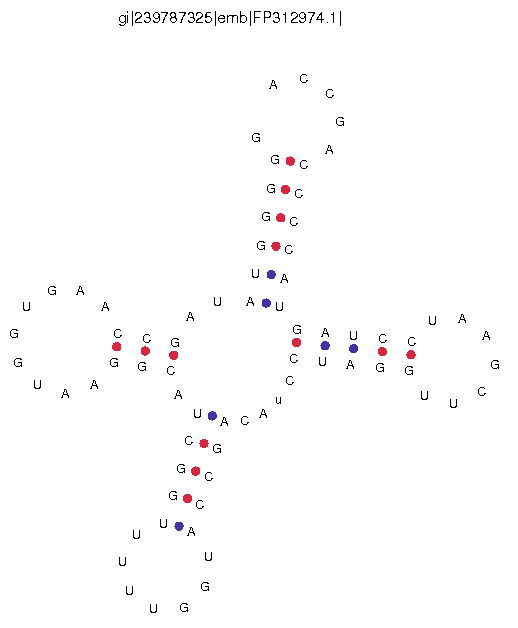 | 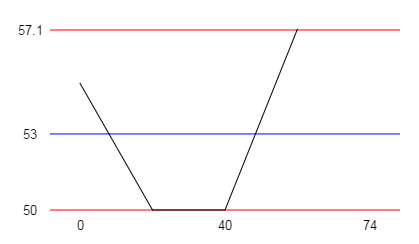 |
| 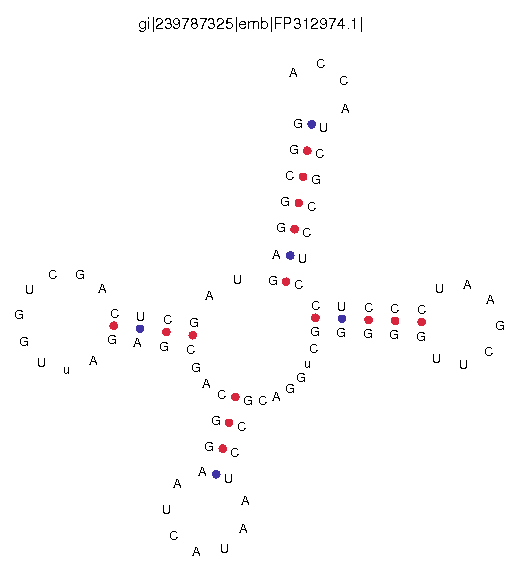 | 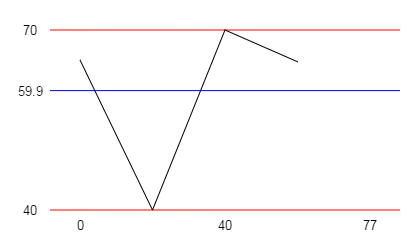 |
| Uncultured bacterial clone magm9502ao02 (FP312975) | |
| 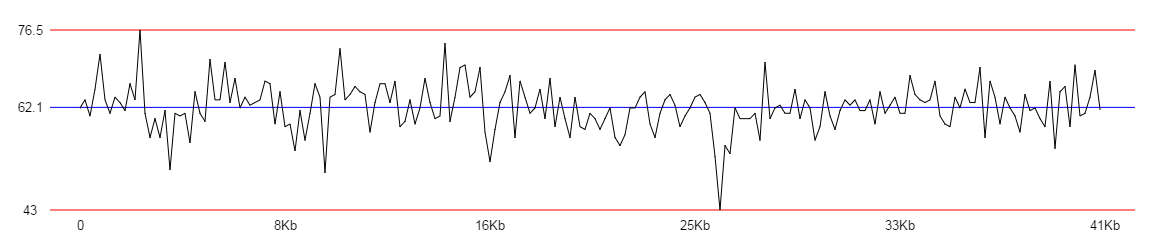 | |
| 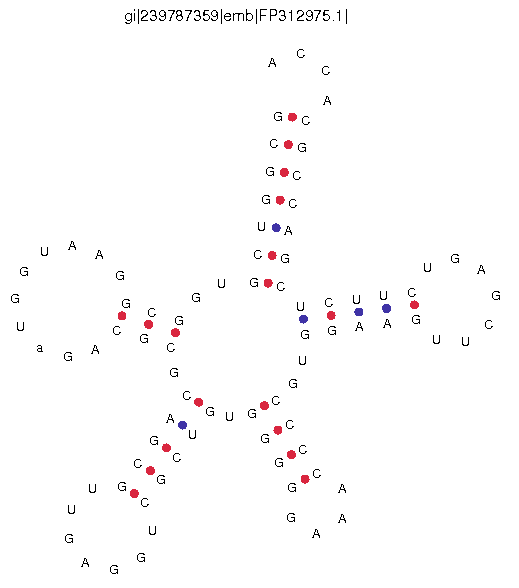 | 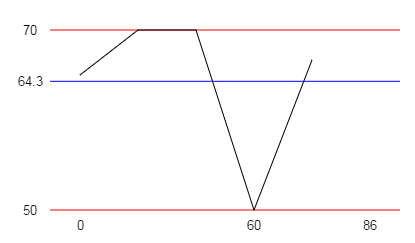 |
| Uncultured bacterial clone mtbe218 (FP312977) | |
| 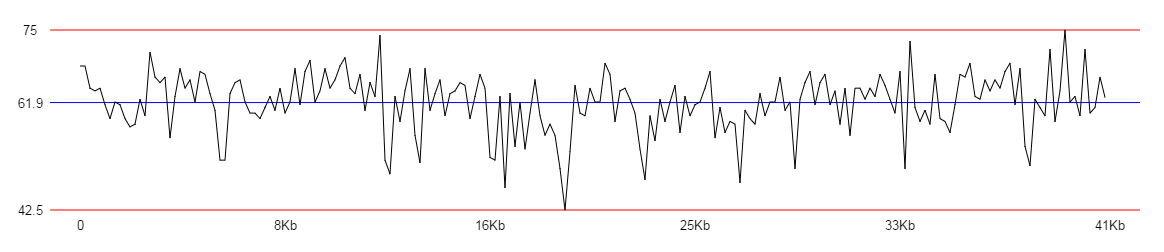 | |
| 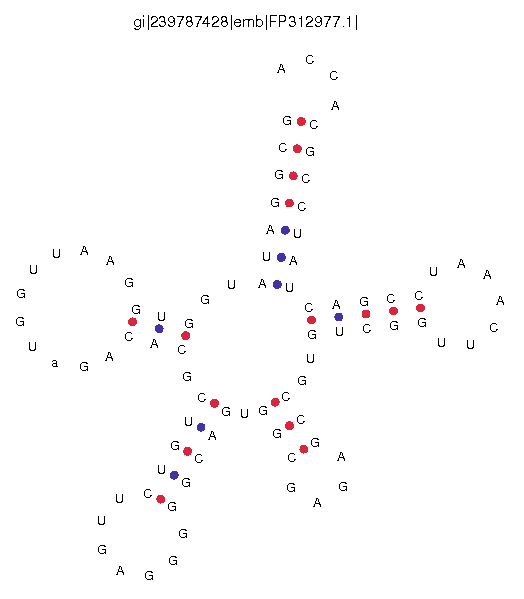 | 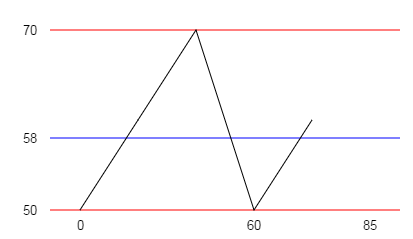 |
| Uncultured bacterial clone mtbm116 (FP312985) | |
| 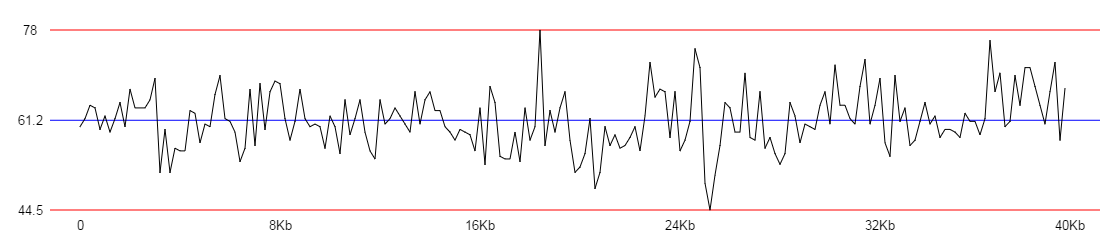 | |
| 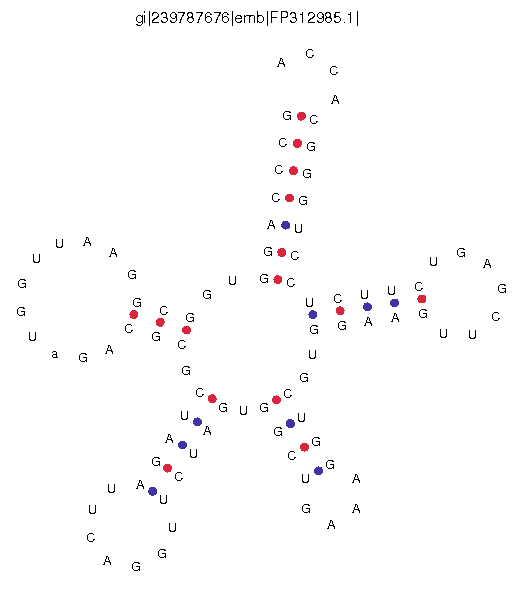 | 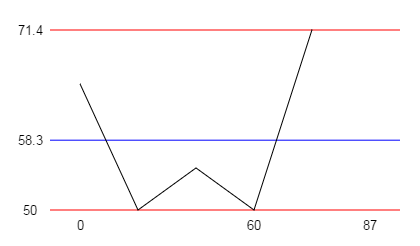 |
| Oscillibacter sp. ER4 contig_75 (NZ_JPJG01000067) | |
| 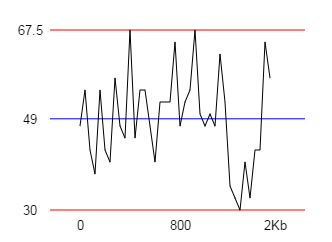 | |
| 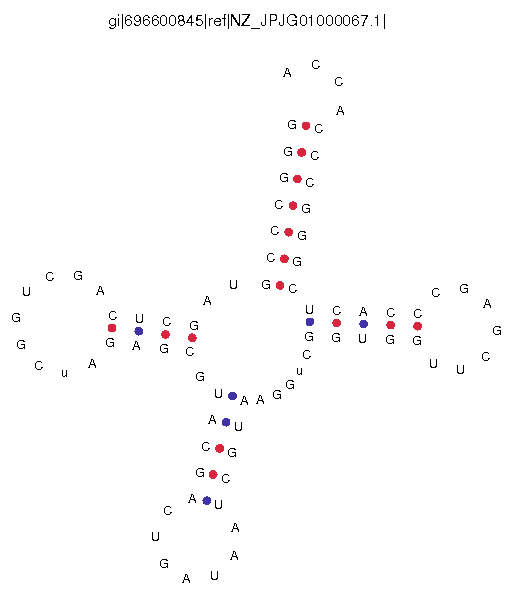 | 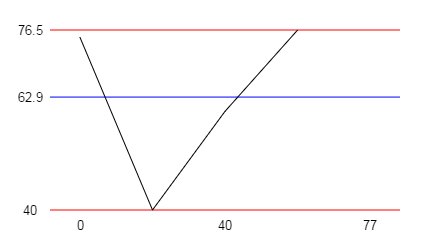 |

Fig. 1 Results of Analysis of tRNA detected in uncultured archaea genomes using tRNAScan-SE tool
